# Applications of Supramolecular Polymers Generated from Pillar[*n*]arene-Based Molecules

**DOI:** 10.3390/polym15234543

**Published:** 2023-11-27

**Authors:** Xu Li, Yan Jin, Nansong Zhu, Long Yi Jin

**Affiliations:** Department of Chemistry, National Demonstration Centre for Experimental Chemistry Education, Yanbian University, Yanji 133002, China13331508107@163.com (Y.J.)

**Keywords:** pillar[*n*]arene, supramolecular polymer, supramolecular interactions, application

## Abstract

Supramolecular chemistry enables the manipulation of functional components on a molecular scale, facilitating a “bottom-up” approach to govern the sizes and structures of supramolecular materials. Using dynamic non-covalent interactions, supramolecular polymers can create materials with reversible and degradable characteristics and the abilities to self-heal and respond to external stimuli. Pillar[*n*]arene represents a novel class of macrocyclic hosts, emerging after cyclodextrins, crown ethers, calixarenes, and cucurbiturils. Its significance lies in its distinctive structure, comparing an electron-rich cavity and two finely adjustable rims, which has sparked considerable interest. Furthermore, the straightforward synthesis, uncomplicated functionalization, and remarkable properties of pillar[*n*]arene based on supramolecular interactions make it an excellent candidate for material construction, particularly in generating interpenetrating supramolecular polymers. Polymers resulting from supramolecular interactions involving pillar[*n*]arene find potential in various applications, including fluorescence sensors, substance adsorption and separation, catalysis, light-harvesting systems, artificial nanochannels, and drug delivery. In this context, we provide an overview of these recent frontier research fields in the use of pillar[*n*]arene-based supramolecular polymers, which serves as a source of inspiration for the creation of innovative functional polymer materials derived from pillar[*n*]arene derivatives.

## 1. Introduction

Supramolecular chemistry, often called “chemistry beyond the molecule”, centers on non-covalent interactions such as hydrogen bonding, host–guest recognition, metal coordination, π–π interaction, cation–π interaction, electrostatic binding, salt bridge, halogen bonding, and so on [1,2]. Self-assembly, a practical technique for designing and controlling materials at the nanoscale, has emerged as a powerful synthetic tool, offering control over attributes like shape, size, geometry, topology, and morphology. Developing advanced self-assembled systems through functional building blocks and “weak” intermolecular and intramolecular interactions has garnered increasing attention since 2005.

Generally, supramolecular polymers represent a fusion of supramolecular chemistry and polymer chemistry, wherein small molecules or polymers are joined through non-covalent interactions creating assemblies with defined structures or functions [3,4]. Diverse units and architectural elements can come together through supramolecular interactions to create supramolecular polymers endowed with unique functionalities. Expanding the range of new supramolecular interactions promises to enhance both the structures and the applications of these supramolecular polymers [5]. Moreover, the process of creating inclusions based on supramolecular interactions can be dynamically regulated by external stimuli, enabling the fabrication of controllable and responsive supramolecular materials with specifically desired characteristics [6]. To date, supramolecular polymer materials have found applications across a diverse range of fields. 

**Scheme 1:**
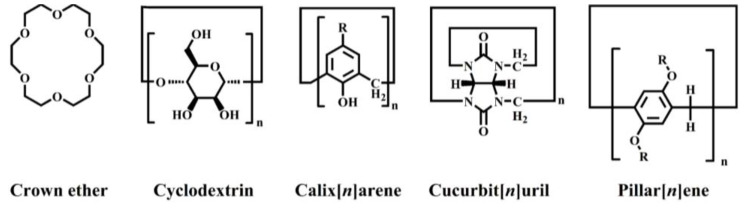
Molecular structures of five different macrocyclic hosts.

In many supramolecular polymer structures, macrocyclic host molecules possess appealing structural attributes, encompassing a hydrophobic cavity, sites for ion binding, versatile yet precisely defined conformations, synthetic adaptability, and adjustable functionality [7,8]. Macrocyclic molecules are used in an extremely wide range of applications, including molecular recognition, analyte detection, drug release, self-healing materials, and artificial nanochannels. Chemical structures of macrocyclic supramolecular polymers can be classified into five types (Scheme 1). Some previous reviews are mainly focused on the designs and applications of the first four generations of macrocyclic supramolecular polymers [9,10,11,12,13,14,15,16], which inspires us to write this review concerning the recent developments of applications of supramolecular polymers generated from pillar[*n*]arene-based molecules. 

Pillar[*n*]arene represents a next-generation macrocyclic molecule characterized by its robust complexation capabilities based on supramolecular interactions, which shows outstanding dynamic and modular performance attributes [17,18], especially in sensing [19,20] and catalytic activity [21,22]. Pillar[*n*]arene was first synthesized by Ogoshi in 2008. Once this unique structure had been discovered, it quickly became a research hotspot [23,24,25,26,27,28,29]. 

The structures and properties of pillar[*n*]arene are quite different from previously reported macrocyclic molecules: (i) Compared with crown ether and calixarene, pillar[*n*]arene has a highly symmetrical rigid structure with hydrophobic cavities at the top and bottom edges. The adjustment of cavity size also has a higher degree of freedom, which shows stronger abilities to identify guests and incorporate catalytic units. (ii) Pillar[*n*]arene has good solubility in organic solvents, which is unique in the main macrocyclic molecules with cavities of the same size. (iii) Compared with cucurbituril, the benzene ring of pillar[*n*]arene is more easily functionalized by different substituents, which can regulate the host–guest binding characteristics more effectively. As a result, a large number of derivatives with unique properties have been obtained. (iv) Pillar[*n*]arene can be an amphiphilic molecule with hydrophobic and hydrophilic groups. A structure with such characteristics can form self-assembly structures in both water and oil phases, which has potential applications in controlled release, gene transfer, photoelectric materials, etc. (v) Pillar[*n*]arene can respond to many external stimuli, including temperature, pH, redox, enzyme, etc., especially light stimulation. These stimuli are easy to operate and can be utilized in a non-invasive and lower-cost manner. This is also one of the advantages of pillar[*n*]arene in sensing compared with other macrocyclic structures.

**Scheme 2:**
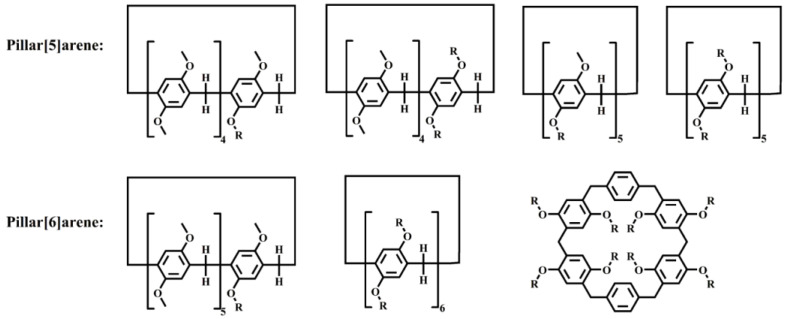
Typical molecular structures of pillar[*n*]arenes discussed in this review. R represents different functional groups, and the detailed structures of R can be found in the following sections.

At present, the mainstream work of pillar[*n*]arenes is focused on prominent pillar[5]arenes and pillar[6]arenes, as shown in Scheme 2. The most commonly studied structure was pillar[5]arenes with a cavity size of approximately 4.7 Å and an average bond angle of 111.3°. The structures of pillar[*n*]arenes of other sizes have not been systematically studied. The production yields of high-level pillar[*n*]arenes (where *n* is equal to or greater than 6) typically remain below 1%, resulting in their infrequent utilization in subsequent investigations. Only a few papers have been reported on pillar[4]arene [30] and pillar[10]arene [31], so this review does not give a specific introduction to them.

The electron-rich cavities of pillar[*n*]arene can form complexes with various guest molecules exhibiting neutral architectures or positive charges ascribed to supramolecular interactions. Guest molecules consisting of long carbon chains generally require cyanide, bromine, or quaternary ammonium salt as the end groups for host–guest identifications. According to the role of pillar[*n*]arenes in the construction of polymer materials, pillar[*n*]arene-based supramolecular polymers (PSPs) are mainly classified into two categories in the discussion of this paper: (i) Pillar[*n*]arene firstly generates complexes with other guests, and such complexes are further assembled to form one-dimensional, two-dimensional, and three-dimensional spatial structures through the effects of non-covalent bonding such as hydrogen bonding, metal–ligand bonding, and π–π stacking [32,33,34,35]; (ii) Pillar[*n*]arene is treated as a kind of functional unit and introduced into the classical polymer substrates through covalent bonding, host–guest interactions, and other non-covalent dynamic interactions [36,37]. Based on the definition of PSPs, the PSP materials discussed in this paper are unique in the following three points compared with the classical covalent macromolecules. Firstly, the main mechanism of supramolecular polymer formations is self-assembly. Secondly, the supramolecular polymers are able to introduce a variety of groups through host–guest recognitions or other weak interaction forces. Thirdly, pillar[*n*]arene-based molecules can self-assemble into chiral supramolecular polymers [38,39,40,41,42]. The constructed supramolecular polymers are a type of tunable material with features such as structural and mechanical integrity, good processability and recyclability, stimuli-responsiveness, self-healing, and shape memory, which holds outstanding advantages over classical covalent macromolecule polymers. In conclusion, PSPs not only inherit numerous advantages of conventional covalent polymers but also offer distinct characteristics [43]. 

Pillar[*n*]arene has been widely used in a variety of scopes ascribed to the structural symmetry, easy synthesis, and functionalization with different substituents on every benzene ring. The exceptional attributes of PSPs have led to their extensive utilization in areas such as sensor arrays [44,45,46,47], substance separation materials [48], catalysis, light-harvesting systems, controlled drug release, as well as membrane delivery and various other domains [49,50,51,52,53,54,55,56]. Some reviews about the designs and applications of PSPs have been reported earlier, especially related to pillar[5]arene (Table 1). However, a comprehensive summary on novel applications of both pillar[5]arene and pillar[6]arene is still lacking. Therefore, in this review, we carefully select and systematically summarize the representative achievements in the molecule designs of PSPs and their six application domains (i.e., fluorescence sensors, substance adsorption and separation, catalysis, light-harvesting systems, artificial nanochannels, and drug delivery systems), intending to advance further breakthroughs in the applications of supramolecular polymers generated from pillar[*n*]arene-based molecules.

## 2. Applications of PSPs

### 2.1. Fluorescence Sensor

**Scheme 3:**
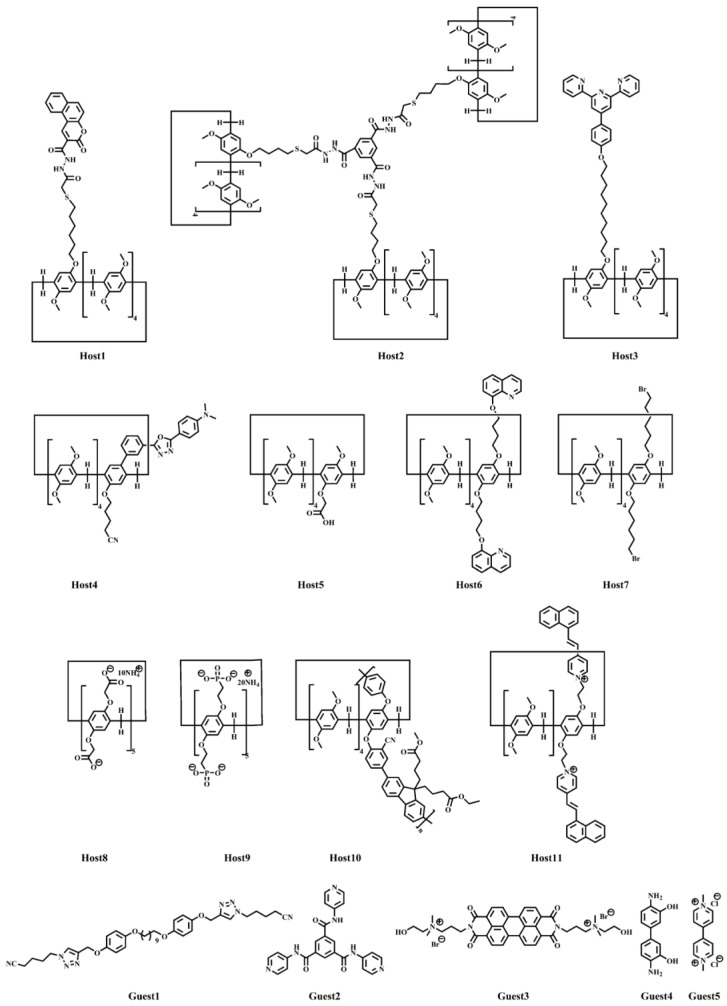
Molecular structures of pillar[*n*]arenes and guest molecules mentioned in this section.

As a novel sensing approach, supramolecular sensors have gained significant attention for their remarkable affinity towards a wide range of analytes. Pillararenes and their derivatives, with macrocyclic cavities, exhibit robust recognition capacities based on supramolecular interactions, particularly for ions and biomolecules, making them a focal point in this burgeoning field. Structure modifications can be achieved using various methods, including thermal responsiveness [65,66,67], electrochemical responsiveness [68,69,70], redox reactivity [71], and the incorporation of fluorescent [72,73,74,75,76,77,78,79,80] and other functional units [81,82,83,84,85,86,87,88,89]. The chemical structures of pillar[*n*]arenes and guest molecules mentioned in this section are listed in Scheme 3. In addition, the detailed properties of fluorescent sensor materials are summarized in Table 2.

Among the numerous response mechanisms available, fluorescence-based sensors have become notable due to their simple synthesis, real-time analysis, high temporal and spatial resolution, reversibility, and exceptional selectivity and limit of detection (LOD) towards analytes [90,91,92,93,94]. 

**Table 2:** Summary of fluorescence sensor materials described in this review.

Type of Pillar[*n*]arenes	Guest	Supramolecular Interactions	Morphology	Application	Ref.
**Host1**	**Guest1**	Host–guest, π–π, Hydrogen bond	Cross-linked 2D networks	Detection and separation of Fe^3+^. Detection type: turn-off. *λ*_ex_/*λ*_em_: 365 nm/440 nm. LOD: 3.0 mM. Linear range: 0–23.3 μM.	[95]
**Host2**	**Guest2**	Host–guest, π–π, Hydrogen bond	Cross-linked network	Ultrasensitive detection and separation of Fe^3+^, Cr^3+^, Tb^3+^, Eu^3+^, etc. Detection type: turn-on/off. *E*_x_ = 467 nm. Linear range: 0−1.8 μM.	[96]
**Host3**	**Guest1**	Host–guest, Coordination	Cross-linked network	Base-responsive convenient test kit for detecting OH^−^ anions. *λ*_ex_/*λ*_em_ = 365 nm/460 nm.	[97]
**Host4**	−	Coordination	Brush	Detection of Cu^2+^ in chloroform. Detection type: turn-off. *λ*_ex_/*λ*_em_: 310 nm/411 nm. Linear range: 0−78 mM.	[98]
**Host5**	−	Coordination	Nanorod	Detection of Fe^3+^, acetone, and nitrophenols in H_2_O. Detection type: turn-off. *λ*_ex_/*λ*_em_: 285 nm/330 nm. LOD: 0.77 μM (*o*-NP); 0.30 μM (*m*-NP); 0.31 μM (*p*-NP); 0.37 μM (TNP). Linear range: 0–500 μM (Fe^3+^).	[99]
**Host6** **Host7**	−	π–π, Coordination	Lamellar stacking	Ultrasensitive response chemosensors, test kits, and fluorescent materials for Fe^3+^ and F^−^. Detection type: turn-off (Fe^3^); turn-on (F^−^). LOD: 0.102 nM (Fe^3^); 9.79 nM (F^−^).	[100]
**Host8**	**Guest3**	Host–guest	Regular block aggregation	Reversible detection of Fe^3+^ in H_2_O. Detection type: turn-on. *λ*_ex_/*λ*_em_: 490 nm/550 nm. LOD: 2.13 mM. Linear range: 0.04−0.30 mM.	[101]
**Host9**	**Guest4**	Host–guest	Irregular loose agglomerates	Water-soluble and good adaptability sensor for Au^3+^ detection. Detection type: turn-off. *λ*_ex_/*λ*_em_ = 290 nm/420 nm. LOD: 69 nM. Lnear range: 0−50 μM.	[102]
**Host10**	**Guest5**	Host–guest	Linear main chain	Recognition of paraquat with high FL quantum yield. Detection type: turn-off. *λ*_ex_/*λ*_em_: 290 nm/430 nm. LOD: 16.9 nM. Linear range: 0−100 μM.	[103]
**Host11**	**Guest5**	Host–guest	Spherical sparse	Molecular recognition of paraquat and bioimaging. Detection type: turn-off. *λ*_ex_/*λ*_em_: 375 nm/527 nm. LOD: 0.706 mM. Linear range: 0.2−5 equiv.	[104]

PSPs can capture specific analytes, leading to observable fluorescence signals that manifest as quenching, fluorescence enhancement, or shifts in response to energy transfer, charge transfer, electron transfer, and strong coordination interactions. Functionalized pillar[*n*]arene holds promising potential for use and advancement in fluorescence sensors. These systems respond to a broad spectrum of potential analytes, encompassing metal cations, anions, drugs, pesticides, and other biomolecules. This section explores constructing fluorescence recognition and detection assays utilizing PSPs.

Metal ions and amino acids are essential components within the fields of chemistry, biology, and the environment. The selective identification and testing of specific analytes and the isolation of toxicity are critical endeavors. In addressing these challenges, numerous research teams have designed and synthesized supramolecular polymers featuring diverse morphologies, including cross-linked networks and brush architectures. Detecting and separating hazardous metal ions like Fe^3+^ are crucial in environmental protection and healthcare.

In 2023, Li et al. [95] introduced a stimuli-responsive supramolecular polymer network to address this challenge. The pillar[5]arene derivative **Host1** engaged in supramolecular interactions with **Guest1**. This innovative configuration displayed impressive fluorescence characteristics attributed to the Aggregation-Induced Emission (AIE) effect and demonstrated remarkable ion-sensing capabilities in both solutions and solid states. Supramolecular polymers have the potential to undergo a transition into supramolecular gels under specific conditions. When the polymer is concentrated, it forms a polymer gel that responds to changes in temperature, mechanical force, and the competitive agent. This xerogel effectively removes Fe^3+^ from water. Additionally, a fluorescence test kit utilizing an ion-responsive film can be conveniently employed for detecting Fe^3+^.

While it is known that AIE materials based on the supramolecular organic frameworks (SOFs) possess the ability to adjust emission, it is worth noting that there are relatively few reports on stimuli-responsive soft materials with the capacity for tunable AIE. Lin et al. [96] developed an AIE supramolecular organic framework gel (SOF-TPN-G) to detect and separate various metal ions. This innovative material allows for recognizing multiple metal ions through the changes in fluorescent signals. **Host2** and **Guest2** join together to create supramolecular polymer networks (SPNs). TPSN serves as the π–π interactions, C−H···π interactions, and inclusion interactions. The thioacetylhydrazine moiety functions as the binding site for hydrogen bonding and coordination. The introduction of certain ions into SOF-TPN-G led to the creation of numerous metallogels exhibiting multicolor fluorescence. Leveraging these gels, an eight-unit sensor was successfully developed, with some units achieving an ultrasensitive performance. The xerogel form of SOF-TPN-G proved highly effective in adsorbing multiple metal ions. These gel materials have significant potential for applications in detection and separation processes.

**Figure 1:**
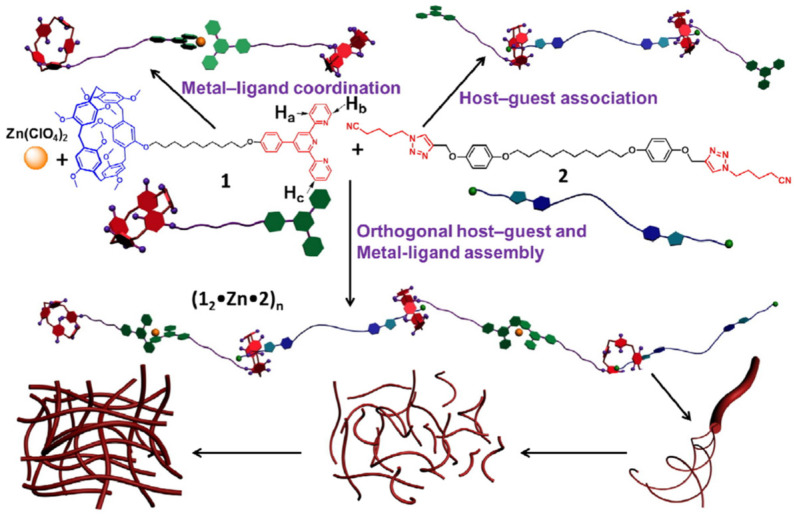
Representation for the formation of a fluorescent supramolecular polymer Co-P[5]Flu constructed by pillar[5]arene-based molecular recognition and zinc ion coordination [97]. Copyright 2015, The Royal Society of Chemistry.

Recent reports indicate that the neutral **Guest1**, featuring a cyano site and a triazole site, has proven to be an efficient guest molecule for **Host3** in supramolecular interactions. Inspired by the stable coordination compounds formed by terpyridyl groups and zinc ions, Yao et al. [97] designed a high molecular weight and fluorescent supramolecular polymer with a cross-linked network (Figure 1). This polymer was created through the principles of molecular recognition and metal coordination involving pillararene. Three-dimensional networks were formed through the entanglement of the supramolecular bundles, and phase transitions were achieved by alternating heating and cooling. The polymer displayed concentration-dependent fluorescence and was responsive to basic stimuli. A thin film was subsequently prepared, which is a convenient test kit for detecting OH^−^ anions. 

In recent years, 1,3,4-oxadiazole (OXD) derivatives have found widespread utility in various domains owing to their exceptional photoluminescent quantum yield and chemical stability.

In collaboration with Zhang and his research team, Han [98] developed a supramolecular brush polymer named Poly(P5-OXD) through the self-assembly of **Host4**, featuring a 1,3,4-oxadiazole unit and a cyanobutoxy group. This polymer exhibited responsiveness to Cu^2+^ ions, resulting in an ON-OFF fluorescent switch. Upon adding Cu^2+^, the supramolecular brush polymers displayed altered structural characteristics, as evidenced by fluorescence quenching, suggesting the potential transformation into cross-linked supramolecular networks.

Over the past decade, AIE has grown significantly across numerous domains. The collaboration between supramolecular materials and coordination chemistry holds immense potential to enhance the rapid advancement of multifunctional materials further. Creating fluorescent coordination polymers incorporating supramolecular macrocyclic rings is a novel and complex research area. In this context, pillarenes are regarded as organic linkers due to their inherent porous properties. In 2018, Wu and Yang [99] developed a fluorescent coordination polymer named CP5-PCP. This polymer featured dicarboxylic acid-containing copillar[5]arene (**Host5**) as the organic linker and Cr^3+^ as the nodes. This sophisticated fluorescent material found applications in detecting Fe^3+^, acetone, and nitrophenols. The creation of this synthetic material has significantly contributed to the advancement of both supramolecular materials and coordination chemistry. 

Recently, there has been a growing focus on ultrasensitive response materials because of their remarkable sensitivity to target substances. Furthermore, the ability to detect exceedingly low concentrations of targeting analytes in environmental or biological systems holds significant importance. Fe^3+^ is the most prevalent and extensively used metal ion in living organisms, ranking among the most critical transition metal ions. Nonetheless, a deficiency and an excess of Fe^3+^ can lead to severe biological disorders and disruptions in normal bodily functions, resulting in diseases. Fluoride ions (F^−^) naturally occur in the environment and are present in the human body. However, excessive fluoride levels lead to environmental contamination and pose health risks, including kidney problems, acute stomach issues, and conditions like dental and skeletal fluorosis. Hence, there is a pressing need to devise novel and straightforward ultrasensitive methods for detecting Fe^3+^ and F^−^ ions. Zhang et al. [100] significantly addressed this challenge by successfully developing a supramolecular organic framework gel PQPA. They achieved this by utilizing bilateral 8-hydroxyquinoline-modified pillar[5]arene (**Host6**) and bilateral bromohexyl-functionalized pillar[5]arene (**Host7**) through supramolecular interaction binding. The LOD achieved by PQPA for Fe^3+^ was an impressive 0.102 nM, highlighting this gel’s selective and ultrasensitive properties. Notably, PQPA proved effective for the detection of Fe^3+^ in real-life samples. Simultaneously, by introducing Fe^3+^ into PQPA gel, a non-fluorescent Fe^3+^ coordination metal gel, PQPA-Fe, can be created. This gel exhibited a “turn-on” fluorescent response to F^−^ due to competitive coordination. The LOD for F^−^ achieved by PQPA-Fe was 9.79 nM. Furthermore, the PQPA xerogel demonstrated the ability to efficiently remove Fe^3+^ at lower concentrations with a high adsorption rate.

Organic solvents are undesirable for ion detection in biological and environmental systems. Consequently, there is significant importance in developing water-soluble derivatives that exhibit exceptional detection capabilities in aqueous solutions.

The sensitivity of perylene diimide derivatives was constrained due to their inherent aggregation-induced quenching (ACQ) properties in aqueous environments. In 2017, Yin et al. [101] introduced a supramolecular interaction system that relied on **Host8** and a quaternized perylene diimide **Guest3** designed for use in aqueous media. In this process, the hydrophobic effects and electrostatic interactions played pivotal roles. In an aqueous solution, **Guest3** formed strong fluorescent aggregates. However, when **Host8** was introduced into the **Guest3** aqueous solution, a change in morphology occurred, leading to a fluorescence “turn-off” phenomenon due to the photoinduced electron transfer (PET) process. The addition of Fe^3+^ reversed this supramolecular PET process, resulting in a “turn-on” fluorescent response. The detection of Fe^3+^ exhibited specific, ratiometric, and reversible behavior, with an LOD of 2.13 × 10^−7^ M. 

Inclusion compound probes that are built using macrocyclic compounds have garnered significant research interest and can detect various metal cations sensitively. Yang et al. [102] introduced a highly sensitive and selective fluorescence sensor capable of quantitatively analyzing Au^3+^ ions in aqueous systems. This sensor achieved an LOD as low as 6.9 × 10^−8^ M. The sensor relies on the supramolecular interactions between **Guest4** and **Host9**, forming an inclusion complex prepared using the saturated solution method. Including **Guest4** by **Host9** significantly increased the water solubility of **Guest4**, boosting it by 177-fold. The inclusion complex IC exhibited a larger Stokes shift in aqueous conditions alone. The stoichiometric ratio between **Host9** and **Guest4** was determined to be 1:1, and the binding constant was calculated to be (4.62 ± 0.15) × 10^4^ M^−1^. Notably, the IC displayed robust resistance to interference and demonstrated excellent adaptability over a wide range of pH values.

The PSPs find extensive applications in drug detection. Investigations were also focused on studying another typical pesticide, namely paraquat (PQ). PQ is a non-selective and fast-acting herbicide that, if accidentally ingested, can severely damage human lungs, liver, kidneys, and other vital organs. Given the significant clinical and environmental implications of PQ, there is a pressing need to design new methods for its high-sensitivity and accurate detection. PQ can interact not only with electron-rich molecules but also form supramolecular interaction complexes and columnar structures with macrocyclic molecules like calixarenes, cucurbiturils, and others. 

Fluorene possesses a planar and rigid biphenyl structure with a high fluorescence quantum yield. To enhance the solubility of the material, chemical modifications were performed on fluorene derivatives by introducing functional side chains at the C9 position of the fluorene molecule. Covalent-bonded conjugated polymers exhibited a molecular wire effect that enhances the response signal in fluorescence sensing applications. 

Building on the principles mentioned earlier, Li, along with Xiao and their team [103], synthesized a novel conjugated polymer named (Co-P[5])Flu. This polymer was obtained through the copolymerization of a difunctionalized pillar[5]arene (**Host10**) and a fluorene derivative monomer. The intramolecular rotations of the pillar[5]arene unit were constrained in Co-P[5]Flu, resulting in an AIE enhancement (AIEE) effect. Pesticide PQ (paraquat, **Guest5**) could enter the pillar[5]arene cavity within Co-P[5]Flu. In EtOH/H2O, Co-P[5]Flu exhibited fluorescence quenching in response to PQ, attributed to the synergistic effects of polypseudorotaxane formation and PET. Co-P[5]Flu demonstrated exceptional selectivity and sensitivity in detecting PQ, with a low LOD of 1.69 × 10^−8^ M and a determined Stern–Volmer constant (*K*_sv_) of 2.11 × 10^4^ M^−1^. This polymer chemosensor proved to be valuable for the practical detection of PQ.

Researchers are actively designing and synthesizing materials with improved performance for PQ detection to enhance the sensitivity of detection. The combination of two different macrocyclic compounds in such efforts holds significant promise and is an intriguing avenue of exploration. Indeed, there has been limited research on the potential applications of such materials thus far. 

**Figure 2:**
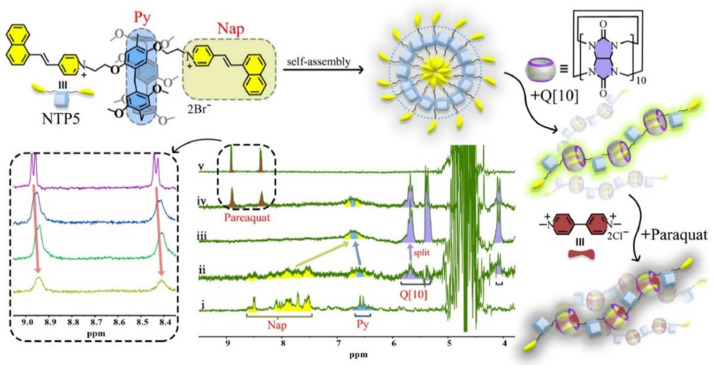
Schematic representation of a novel pillar[5]arene-cucurbit[10]uril−based complex and its application for the detection of paraquat. Illustration (top) of the interactions of NTP5, Q[10]-NTP5, Q[10]-NTP5-PQ, and the 1H NMR spectrum (bottom) (inset: Q[10]-NTP5 at different concentrations of PQ) [104]. Copyright 2023, Wiley publishing.

However, a new category of supramolecular polymers involving two distinct macrocyclic host molecules, namely pillar[*n*]arene NTP5 (**Host11**) and Q[10], was successfully synthesized by Xiao et al. [104]. Representation for the formation of a fluorescent supramolecular polymer was shown in Figure 2. The inclusion of the macrocyclic Q[10] significantly positively impacted the fluorescence properties of NTP5 by constraining its intramolecular rotation. Specifically, the 4-[2-(1-naphthalenyl)ethenyl]pyridine moiety of NTP5 was encapsulated by Q[10], leading to the formation of a supramolecular interaction complex. The introduction of Q[10] caused a transformation in the morphology of NTP5, changing it from a spherical structure to a porous one, resulting in enhanced fluorescence. Interestingly, the fluorescence of Q[10]-NTP5 could be quenched by adding PQ, making it suitable for molecular recognition and bioimaging applications.

### 2.2. Substance Adsorption and Separation

**Scheme 4:**
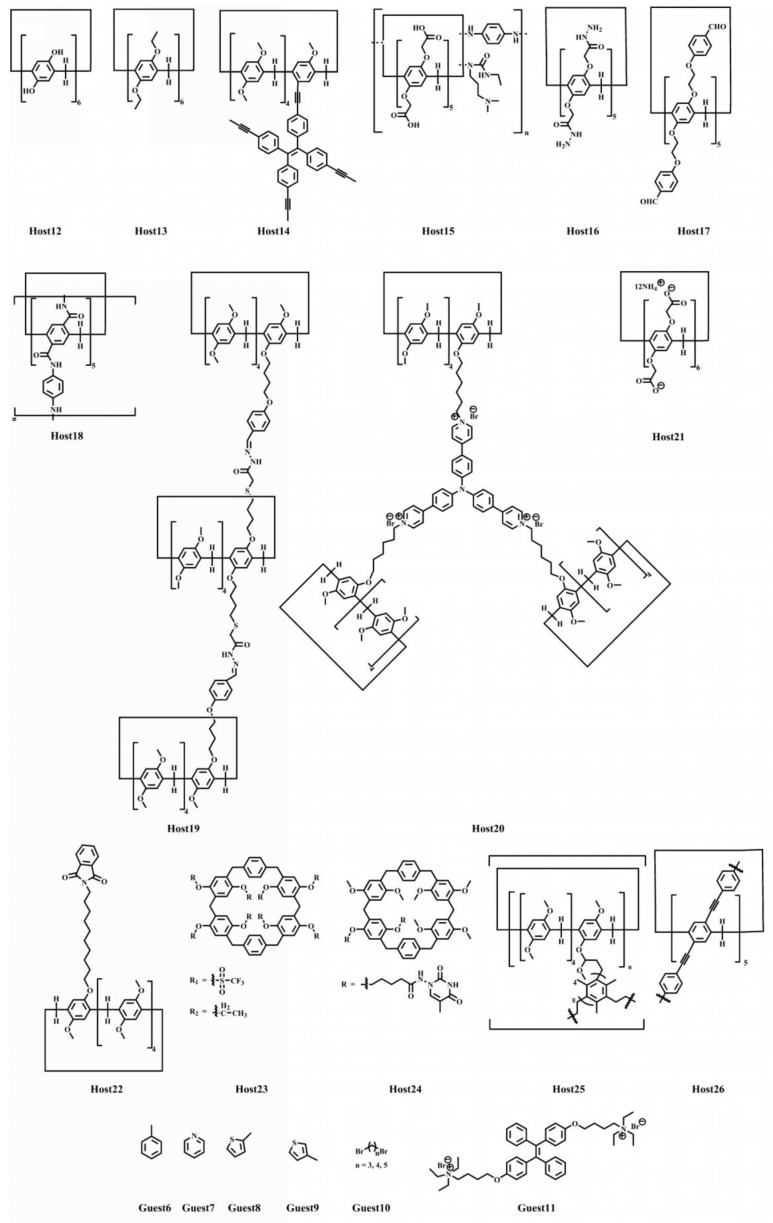
Molecular structures of pillar[*n*]arenes and guest molecules mentioned in this section.

Pillar[*n*]arene offers a diverse range of supramolecular interactions, making it a highly sought-after building block for SPs (supramolecular polymers) with multifunctionality [105,106]. Additionally, the structure of the polymers can create novel voids within the networks and spaces [107], complementing inherent pillar[*n*]arene cavities, which functions as new binding sites for guest molecules [108,109]. Achieving the environmentally friendly and efficient removal of various pollutants is a crucial challenge in environmental science and industrial applications. This issue directly impacts human health and the health of aquatic ecosystems [110,111,112,113,114,115,116]. Pillararene materials serve as protective containers, accurately capturing and safely storing harmful molecules [117,118,119]. The properties and applications of polymers are influenced by factors such as the size of the macrocyclic pore, connectivity, and the chemical environment within the hosts. Self-assembly aggregates contribute to the removal effect. This concept provides a novel approach to adsorbing and separating ions and organic substances. The properties of adsorption and separation materials discussed in this section are listed in Table 3. Meanwhile, the chemical structures of pillar[*n*]arenes and guest molecules mentioned in this section are listed in Scheme 4.

Materials featuring micropores are highly suitable for gas storage and separation applications. A growing focus has recently been on discrete organic molecules instead of 1D-3D networked materials. This shift is driven by the advantages of solubility, flexibility, and the feasibility of obtaining these materials through organic synthesis. Ogoshi et al. [120] found that the aromatic molecule within the pillar[6]arene (**Host12**) forms a highly ordered 1D channel with a diameter of 6.7 Å. This supramolecular assembly channel can capture various gases, including water vapor-saturated hydrocarbons. The organic gas and vapor adsorption capacity of pillar[6]arene in a solid state presents a promising avenue for further exploration.

In industrial processes, toluene is primarily derived from coal and petroleum sources through catalytic reforming, pyrolysis of hydrocarbons, and coking. However, these processes often leave trace amounts of heterocyclic compounds behind, which can significantly impact the properties of toluene. Therefore, efficiently removing these heterocyclic compounds is crucial for obtaining high-purity toluene.

Zhu et al. [121] introduced a separation strategy for mixtures of toluene and heterocyclic compounds using non-porous adaptive guest-loaded crystals of perethylated **Host13**. The approach successfully enhanced the purity of toluene, increasing it from 96.78% to 99%. The stability difference strongly influenced the selectivity of this material in the EtP6 crystal structure after incorporating various guest molecules (**Guest6, 7, 8,** and **9**). Upon removing the guest molecules, the structure of EtP6 reverted to its original state. 

**Figure 3:**
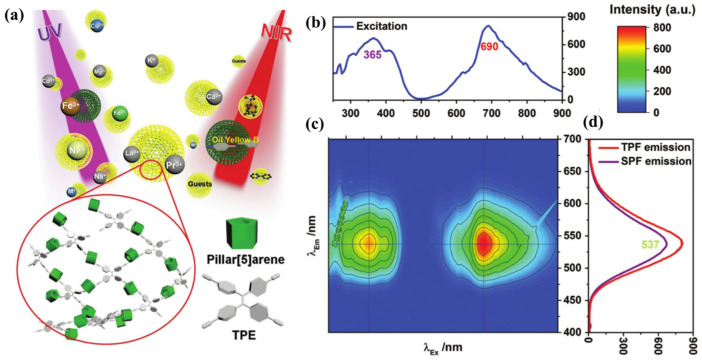
(**a**) Schematic representation of a conjugated macrocycle polymer (P[5]-TPE-CMP) based on pillar[5]arene and TPE and its two-photon sensing for metal ions and organic molecules. (**b**) Excitation spectrum (blue). (**c**) The 3D fluorescence contour spectroscopy of P[5]-TPE-CMP. (**d**) Single-photon fluorescent emission spectrum (purple) and two-photon fluorescent (TPF) emission spectrum (red) [122]. Copyright 2018, Wiley publishing.

Tetraphenylethylene (TPE), which stands for AIE properties, was linked to P[5] using a Sonogashira–Hagihara cross-coupling reaction, resulting in the formation of P[5]-TPE (**Host14**). In their study, Yang et al. [122] introduced a macrocyclic cross-linked reticular polymer, P[5]-TPE-CMP, which demonstrated remarkable properties, including resistance to photobleaching, insolubility, recyclability, and selective sensing capabilities for Fe^3+^ ions and 4-amino azobenzene (Figure 3). P[5]-TPE-CMP also functionalized as a two-photon fluorescence sensor, detecting wavelengths in the 300–400 nm and 650–800 nm ranges. In solution, it exhibited a range of microstructures, including various sizes of spherical and/or rodlike structures. Both P[5]-TPE-CMP materials displayed remarkable thermal stability, remaining stable up to 300 °C in air. The material demonstrated sensitivity and selectivity in the detection process, making it a promising candidate for removing harmful safety-related substances.

Organic micropollutants, including drugs, hormones, pesticides, and industrial chemicals in water systems, threaten human health and aquatic ecosystems. Consequently, creating rapid, efficient, and cost-effective chemical and physical water treatment methods is a pressing challenge for material chemists. The design and synthesis of sustainable adsorbent materials that offer fast adsorption and high absorption capacity remain significant challenges in addressing the removal of organic micropollutants from water. 

In 2017, Shi et al. [123] developed a 3D network polymer known as P5-P (**Host15**), which was cross-linked using carboxyl-derived pillar[5]arene and *p*-phenylenediamine. This polymer exhibited excellent capabilities for adsorbing and removing organic micropollutants from water. Furthermore, this polymer demonstrated recyclable adsorption performance, particularly for fluorescein sodium and methyl orange. The 3D network polymer exhibited the potential for rapid waste-water treatment. Two years later, they applied hydrazide-functionalized pillar[5]arene (**Host16**) and 4-aldehydephenyl-functionalized pillar[5]arene (**Host17**) to obtain another intelligent hydrogel [124]. The hydrogel exhibited a remarkable removal efficiency of 91.5% for organic micropollutants in water. The primary driving force behind its action was the supramolecular interactions between the macrocycle and pollutant molecules. The hydrogel material could be regenerated at least five times by disrupting the supramolecular interaction complex.

The most prevalent sources of water pollution stem from industrial waste discharges and municipal sewage, which release a combination of chemicals and microorganisms. Recognizing the severity of this issue, Wen et al. [125] developed a conjugated polymer based on pillar[5]arene (**Host18**), which is an acid-derivatized poly-*p*-phenylene pillar[4]arene[1]terephthamide (P[5]-PPTA). In P[5]-PPTA, the pillar[5]arene rings engage in supramolecular interactions with short-chain alkyl derivatives. The polymer exhibited a unique absorption capability for low-molecular-weight alkylamines, alcohols, and carboxylic acids in water, and it maintained its performance across five successive cycles of reuse without degradation. Furthermore, P[5]-PPTA could be thermally reactivated, enhancing its versatility for water treatment applications. 

In the studies of separation materials, Wang et al. [126] designed an adsorbent material for PQ by harnessing the combined effect of linear tri-pillar[5]arene-based compounds (**Host19**). This design linked three pillar[5]arene groups within a single acceptor molecule. This material demonstrated several advantages, including a faster adsorption rate for PQ compared to activated carbon and single-pillar[5]arene-based materials. Additionally, its removal efficiency met expectations, with a remarkable 98% adsorption rate for commercial pesticide PQ in water. A year later, Zhang et al. [127] introduced a highly effective tripodal molecule based on tri-pillar[5]arene (**Host20**), which exhibited a “synergistic effect” for the efficient detection and removal of the pesticide PQ through supramolecular interactions. Each molecule featured two adjacent pillar[5]arene groups capable of binding one PQ molecule through multi-non-covalent interactions. The LOD was impressively low, reaching 2.23 × 10^−7^ M. The testing paper utilizing **Host20** could be a detection kit for PQ in water across a wide concentration range (10^−6^–10^−1^ M). Moreover, it demonstrated exceptional adsorption capabilities in water, with an impressive adsorption efficiency of 98.40% for commercial PQ and a removal capacity of 108.24 mg/g. Yang et al. [128] designed and successfully prepared a controllable supramolecular coordination polymer Zn^2+^@WP5 (**Host21**) in a related development. The polymer displayed outstanding adsorption and removal capabilities for PQ, owing to its negative charge, high surface area, and recognition capacity based on supramolecular interactions. The maximum adsorption capacity for PQ reached approximately 203.60 mg/g. The polymer was successfully employed in real sample applications and could be regenerated by exchanging the guest structure.

Anions are crucial in maintaining human health, biological systems, and environmental stability. The precise detection and separation of these ions are of paramount importance. Consequently, the ultrasensitive detection and separation of various environmental ions are gaining significance. Lin et al. [129] developed a smart blue-white AIE gel material (PMDP-G), which was constructed using functionalized **Host17** and **Host22**, utilizing “exo-wall” π–π and supramolecular interactions. PMDP-G demonstrated remarkable sensitivity in detecting and separating multiple analytes, achieved through the interplay between “exo-wall” π–π and cation–π interactions. The low LOD underlined its high sensitivity. PMDP-G exhibited the capacity to adsorb and separate multiple metal ions from aqueous solutions, including Fe^3+^, Hg^2+^, and Ag^+^, with adsorption rates ranging from 90.12 to 99.95%.

Water contamination from heavy metal ions poses a grave threat to humans and animals globally. The development of a comprehensive approach for real-time sensing and the swift removal of mercury(II) ions holds immense significance in materials chemistry, chemical engineering, and environmental science. In recent years, there has been a growing emphasis on designing and synthesizing cost-effective and efficient materials for the adsorption and removal of Hg^2+^ ions. Developing novel technologies for detecting Hg^2+^ in the atmosphere and industrial waste water is essential to enhance environmental protection and safeguard life on our planet.

**Figure 4:**
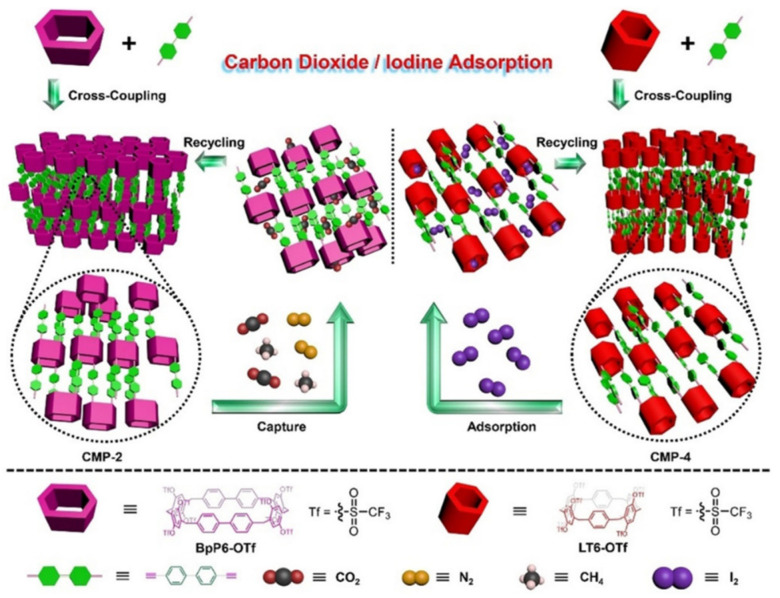
Illustration of the construction of four CMPs (CMP-*n*, *n* = 1–4), selective capture of CO_2_, and efficient adsorption of I_2_ [130]. Copyright 2021, Wiley publishing.

As reported by Yang in 2018, leaning tower[6]arenes (LT6, **Host23**) exhibit a distinctive leaning tower conformation with minimal substituents, which sets them apart from the traditional pillar[6]arenes structures. These molecules have shown excellent performance in supramolecular interaction systems and macrocyclic chemistry. Yang’s group developed a set of four novel CMPs (CMP-*n*, *n* = 1–4) using per-triflate-functionalized and [2]biphenyl-extended pillar[6]arene (Figure 4) [130]. CMP-4 exhibited an exceptional affinity for I_2_, with a vapor uptake capacity of 208 wt % and a remarkable removal efficiency of 94 %. These properties were attributed to the well-suited cavity size of the tower[6]arene and numerous aromatic rings within the framework. CMP-2 displayed no capacity to adsorb N_2_, making it highly selective for CO_2_ capture.

In 2019, Wu et al. [131] introduced a straightforward separation method utilizing a novel type of non-porous adaptive crystals built upon inclined pillar[6]arene (**Host23**). These deoxidized perethyl-inclined pillar[6]arene crystals (EtLP6) displayed non-porous characteristics, exhibiting a preference for 1-bromoalkane isomers over 2-bromoalkane isomers in the separation process. EtLP6 demonstrated the ability to separate 1-bromopropane, 1-bromobutane, and 1-bromopentane from their 1/2 isomer mixtures at a 1:1 (*v*/*v*) ratio in a single adsorption cycle. The obtained purities ranged from 89.6% to 96.3%. This selectivity is due to the distinct supramolecular interaction binding modes and varying stability of EtLP6 crystals loaded with one-site and two-site isomers.

In 2019, Dai et al. [132] introduced a fluorescent supramolecular polymer. This polymer was created using a [2]biphenyl-extended **Host24** with two thymine sites as arms, along with a tetraphenylethylene-bridged bis(quaternary ammonium) **Guest11**. Spherical nanoparticles were generated upon adding Hg^2+^, increasing the emission intensity. This approach was suitable for the real-time detection and removal of Hg^2+^. It demonstrated a rapid response, high selectivity, and fast adsorption rates. Additionally, the material could be recycled by treatment with Na_2_S.

Arunachalam et al. [133] made significant contributions to the removal of iodine vapor by successfully synthesizing a recyclable 3D cross-linked microporous polymer based on pillar[5]arene **Host25**, which demonstrated an iodine vapor capture capability of 3.84 g/g at 80 °C. The polymer adsorbent efficiently removed over 83% of iodine within 15 min of shaking in an aqueous solution. Pillar[*n*]arene holds significant potential for applications in environmental remediation. Additionally, their versatility allows for various possibilities for further functionalization, enhancing their utility in environmental improvement.

To address the issue of water stability in separation systems, Talapaneni et al. [134] introduced an efficient conjugated microporous polymer based on pillar[5]arenes (P5-CMPs, **Host26**). These P5-CMPs were created by attaching triflate-functionalized P5 to 1,4-diethynylbenzene and 4,4′-diethynyl-1,1′-biphenyl linkers. The P5-CMPs exhibited an impressive surface area of up to 400 m^2^∙g^−1^. In earlier studies, porous materials displayed isosteric heats of adsorption (*Q*_st_) for propane at zero coverage in the range of 32.9–36.9 kJ/mol. The *Q*_st_ value increased as the propane loading increased, indicating stronger intermolecular interactions between propane molecules. The *Q*_st_ value reached a maximum of 53 kJ/mol at zero coverage and then gradually decreased to approximately 35 kJ/mol as the loading of propone increased. This phenomenon was attributed to the formation of a robust supramolecular complex between propane and pillar[5]arenes, facilitated by multiple C–H···π interactions. This method introduced a thermodynamic selective separation of saturated hydrocarbons with low polarizability. The strong affinity of P5-CMPs for propane allowed for efficient breakthrough encapsulation and separation from natural gas mixtures under simulated conditions at 298 K.

### 2.3. Catalysis

**Scheme 5:**
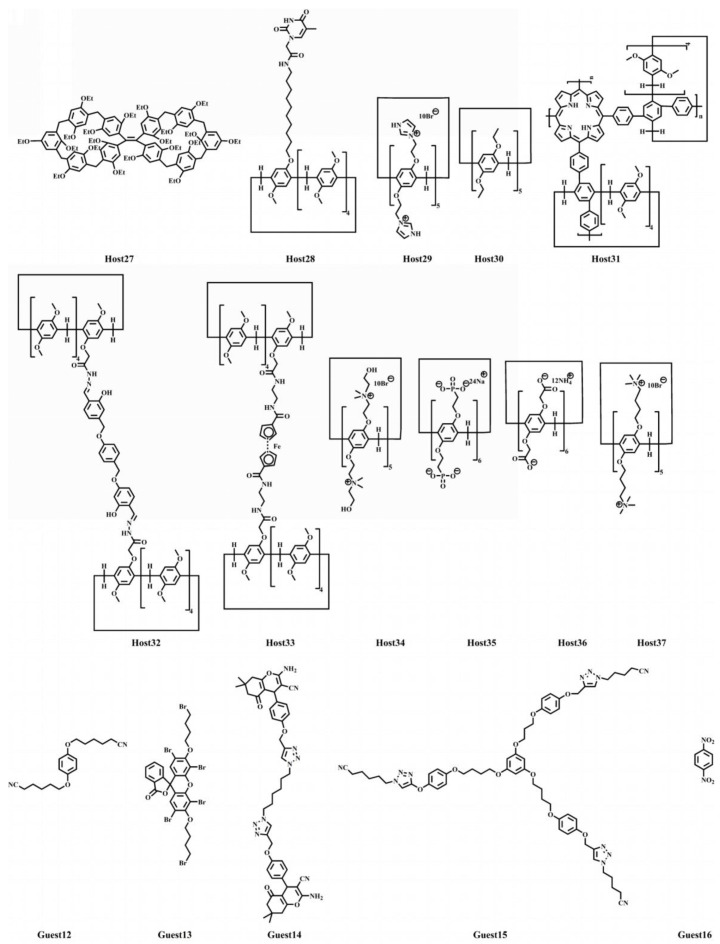
Molecular structures of pillar[*n*]arenes and guest molecules mentioned in this section.

Taking inspiration from nature, supramolecular chemists employ supramolecular macrocyclic systems to mimic the structures and functions of biological enzymes. This allows for supramolecular catalysis by precisely controlling the interactions between host and guest molecules [135,136,137,138,139,140,141]. Materials based on macrocyclic pillararene exhibit unique stability, cyclic structure, and facile separation characteristics. These properties of pillararene can be altered through modification or functionalization, enhancing the chemical, spatial, and stereoselectivity of catalytic reactions [142,143,144,145]. Functionalized pillar[*n*]arene can selectively incorporate catalytic units, creating efficient catalytic sites. The cavity can interact with substrates of appropriate sizes, creating an optimal catalytic environment that enhances reaction efficiency [146,147]. High efficiency and recyclability are crucial for assessing catalyst performance, particularly in industrial production [148,149,150,151,152,153]. This section comprehensively discusses the catalytic applications of pillar[*n*]arene-derived polymers with catalytic activity. Recent catalysts based on PSPs are summarized in Table 4. And the chemical structures of pillar[*n*]arenes and guest molecules mentioned in this section are listed in Scheme 5.

**Figure 5:**
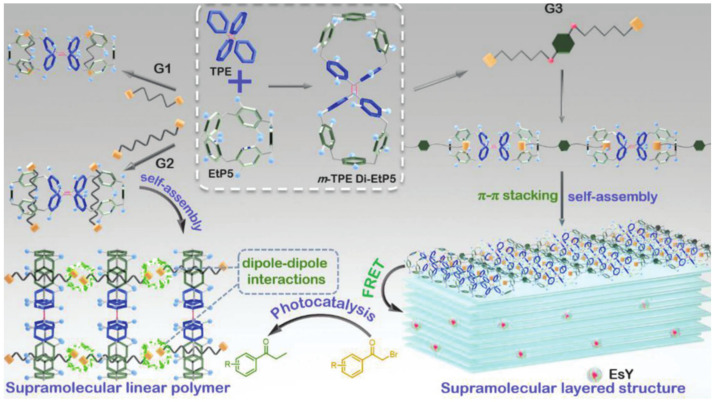
Schematic illustration of the controllable assembly of *m*-TPE Di-EtP5 with guests and the photocatalytic process [154].

To date, two main strategies have been employed for designing luminescent macrocycles. The first approach involves attaching a fluorophore to the edge of the macrocycles, while the second approach focuses on modifying the macrocycles to exhibit AIE properties. The latter approach has recently garnered more attention in developing supramolecular functional materials. As an illustrative example, in 2023, Hu et al. [154] introduced a dimeric fluorescent compound *meso*-tetraphenylethylene dimeric ethoxypillar[5]arene (*m*-TPE Di-EtP5, **Host27**) (Figure 5). Two pillar[5]arenes were linked via a C=C bond, forming a central TPE moiety with AIE properties. This structure exhibited an energy transfer efficiency of 90.3%. The pillararenes also engaged in supramolecular interactions with dinitrile derivatives (**Guest12**). The morphology of these macrocycles could be controlled, and this method of constructing AIE macrocycles offers promise for applications in various fields such as photocatalysis, photodynamic therapy, chemical sensing, and bioimaging.

Indeed, incorporating quantum dots (QDs) and organic dyes into the PSPs can lead to the development of highly catalytic materials. For example, Zhong et al. [155] created luminescent hybrid supramolecular structure light-harvesting systems (LHSs) by directly embedding CsPbBr_3_ quantum dots within a self-assembly of thymine **Host28** and a **Guest13** molecule derived from Eosin Y (ESY). Incorporating CsPbBr_3_ quantum dots and ESY molecules resulted in an efficient energy transfer, achieving a high energy transfer efficiency of 96.5%. This combination exhibited outstanding photocatalytic activity, particularly in cross-coupling hydrogen evolution reactions, where the product yield exceeded 2.5 times that of ESY alone.

In recent times, researchers have made progress in creating and combining hybrid nanomaterials using noble metallic nanoparticles like gold, silver, palladium, and platinum, along with macrocycles. Nanocatalysts based on metal nanoparticles exhibit outstanding catalytic characteristics, influenced by the metal’s surface area and the atoms at its edges or corners. Integrating nanoparticles with macrocyclic hosts makes it possible to greatly amplify the benefits of both components by broadening their potential uses in various technologies such as sensors, drug delivery, and catalysis, among others. 

The Nobel Prize in Chemistry was granted in 2010 to the collective effects of Heck, Negishi, and Suzuki in recognition of their significant contributions to palladium-catalyzed cross-couplings. Subsequently, numerous researchers have become involved in related studies to enhance the solubility, stability, and catalytic activity of the reaction substrates.

In 2018, Shi et al. [156] introduced an economical and straightforward method for synthesizing a heterogeneous catalyst known as a pillar[5]arene-based organometallic cross-linked polymer (P[5]-OCP, **Host29**). This catalyst was obtained by utilizing the complexation of imidazolium-derivative pillar[5]arene with Pd(OAc)_2_. This material offers several advantages, including stability, recyclability, and ease of separation. P[5]-OCP exhibited remarkable catalytic activity in the Suzuki–Miyaura coupling reaction. Due to its recyclability, this catalyst holds significant promise as a candidate in materials science for multiple applications.

Pillar[*n*]arene stands out among various macrocyclic compounds because of its increased structural rigidity and simplified functionalization. [2]Rotaxane, characterized as mechanically interlocked molecules, is an excellent candidate for molecular machines and devices. In 2020, Yao’s team, in collaboration with Yan [157], created a [2]rotaxane utilizing pillar[5]arene (**Host30** and **Guest14**). They formed the organometallic cross-linked catalyst (Pd@R) by coordinating triazole groups with Pd(NO_3_)_2_. The Pd@R catalyst exhibited superior catalytic performance and recyclability in the Suzuki–Miyaura coupling reaction compared to available commercial alternatives. This study contributed to advancing the field of supramolecular polymeric materials to a significant extent. 

Porphyrins possess unique cavities that have shown significant advancements in selective adsorption and pollution mitigation. They have been integrated into the framework of persistent organic pollutants, leading to a spectrum of valuable applications abroad in various domains. Yang et al. [158] introduced a new nanoscale DMP[5]-TPP-CMP, composed of the macrocyclic compound pillararene and porphyrin (**Host31**). These molecules served as alternating strut/node elements. By incorporating PdNPs into CMP (Pd@CMP), researchers achieved successful results, demonstrating exceptional catalytic performance in Suzuki–Miyaura coupling and nitrophenol reduction reactions. The Pd@CMP substance accelerated the Suzuki–Miyaura coupling reaction and significantly enhanced the yield, all while using mild reaction conditions. The *k*_app_ value reached an impressive 1.90 × 10^−2^/s. This recyclable and versatile Pd@CMP material was created as a stable and environmentally friendly catalyst.

One year later, Cai et al. [159] joined the effort to develop environmentally friendly supramolecular polymer catalysts. Their focus was creating a smart hybrid polymeric material through supramolecular interactions and hydrogen bonds. The supramolecular polymer was constructed using a pillar[5]arene dimer (**Host32**) and three-arm **Guest15**. PdNPs were synthesized by introducing Schiff-base groups into a 3D supramolecular polymer. These hybrid supramolecular polymeric materials displayed remarkable catalytic performance in reducing toxic nitroaromatics compounds and facilitating C-C bond-forming Suzuki–Miyaura reactions. 

The structural aspects of our supramolecular polymeric materials comprise ferrocene components, which can trigger the production of **·**OH through a Fenton-like reaction in the presence of H_2_O_2_. Yan et al. [160] synthesized a linear supramolecular polymeric catalyst (**Host33** and **Guest1**). This material demonstrated outstanding catalytic characteristics for the Fenton reaction in water. This material underwent extensive characterization through various methods. At a high concentration of 200 mmol, the polymer additionally self-assembled into supramolecular polymeric networks. Besides its applications in catalysis, the material exhibited significant potential for diverse applications in various fields.

**Figure 6:**
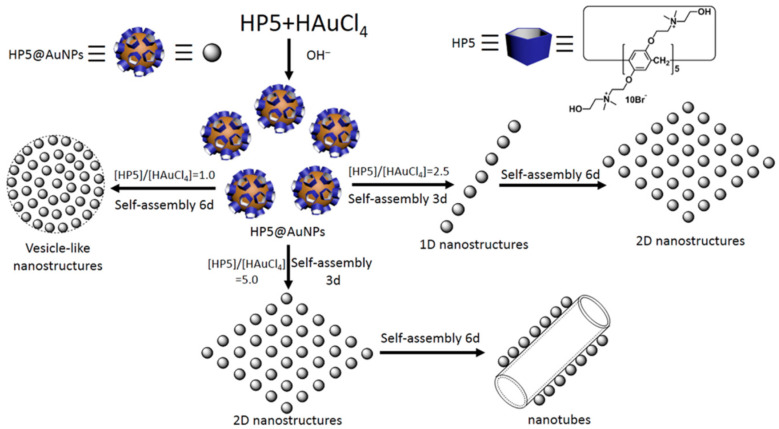
Schematic representation for the formation of HP5−modified AuNPs and their supramolecular self-assemblies [161]. Copyright 2018, American Chemical Society.

In 2018, Zhou et al. [161] produced monodisperse AuNPs through a process involving AuCl_4_^−^ and hydroxylatopillar[5]arene (HP5, **Host34**), relying on supramolecular interactions (Figure 6). These AuNPs exhibited a consistent diameter of 5.0 nm. What is noteworthy is that HP5-functionalized AuNPs could autonomously self-assemble and create structures without requiring a guest mediator. The engineered HP5@AuNPs found applications as scaffolds and energy acceptors in turn-on fluorescence sensors. Notably, these AuNPs displayed remarkable catalytic efficiency in reducing 4-nitrophenol, showcasing their potential versatility in catalysis applications.

Afterwards, Borjigin et al. [162] developed a low-toxicity Au@HP5@SWCNT nanocomposite (**Host34** and **Guest16**) with exceptional catalysis and sensing characteristics. This nanocomposite involved the dispersion of AuNPs on the surface of single-walled carbon nanotubes functionalized with HP5. The process was facilitated at room temperature due to the combined influence of π–π stacking and coordination effects. The outstanding recognition capability of HP5 was the primary factor behind the material’s high catalytic activity in the ethanol oxidation reaction. 

The potential applications of depositing AgNPs onto single-walled carbon nanotubes (SWCNTs) were extensive. Nonetheless, the frequent occurrence of aggregation in SWCNTs, attributed to robust π–π stacking interactions, adversely affected the performance of SWCNTs. Moreover, AgNPs tended to aggregate, which diminished their catalytic activity. Consequently, it was crucial to identify a method or molecule capable of stabilizing AgNPs while simultaneously preventing the aggregation of SWCNTs. To advance the progress of pillar[*n*]arene-based catalysts, Guo et al. [163] produced a nanomaterial Ag@PP6@SWCNT (**Host35** and **Guest5**). AgNPs, with an average size of 3–4 nm, were deposited on the surface of SWCNTs functionalized with PP6 under mild and environmentally friendly conditions. The silver particles coordinated with the PO_3_^2–^ groups within PP6 (**Host35**), and π–π interaction occurred between SWCNTs and the benzene rings of PP6. Compared to commercial catalysts, Ag@PP6@SWCNT exhibited significantly higher activity in the reduction of 4-nitrophenol and the degradation of methylene blue.

In the same year, Tan et al. [164] utilized regular AgNPs to create a 2D heterogeneous hybrid material WP6@Ag@COF (**Host36** and **Guest5**). This material displayed remarkable electrocatalytic activities and electrochemical detection capabilities, resulting in the development of a time-efficient, highly sensitive, and selective electrochemical sensor. The WP6@Ag served as both a host and an electrocatalyst in this setup. It was assembled onto the surface of a covalent organic framework (COF), which was employed to construct an electrochemical sensing platform. This platform could detect PQ within a concentration range of 0.01–50 μM, with a LOD as low as 0.014 μM. The binding constant between WP6 (**Host36**) and PQ was determined to be 1.02 × 10^8^ M^−1^, primarily attributed to the hydrophobic effects and electrostatic interactions. The research holds promise for advancing catalysis studies and applications.

Polyoxometalates are a transition metal oxide anion cluster class with diverse chemical compositions and nanostructures. Wei et al. [165] designed a supramolecular catalyst, PTCs, in which nanospheres co-assembled in aqueous environments. This assembly was achieved by chromium-centered polyoxometalates and cationic pillar[5]arenes (**Host36**). Under enzymatic conditions, this supramolecular catalyst could oxidize aldehydes to enhance conversion rates. Altering the charge ratios of the ionic complexes (P5A-CrMo_6_) led to variations in the diameter of nanospheres. This change resulted from the formation of more compact assemblies as a consequence of closer charge ratios.

### 2.4. Light-Harvesting System

An enduring issue facing humanity is energy scarcity [166,167,168,169,170,171]. Developing effective LHSs (light-harvesting systems) is crucial for addressing the energy crisis and advancing research in photocatalysis, optical sensing, and light-emitting devices [172,173,174,175,176,177,178,179,180]. PSPs exhibit reconcilable and reversible characteristics for the construction of LHSs [181,182,183,184].

**Scheme 6:**
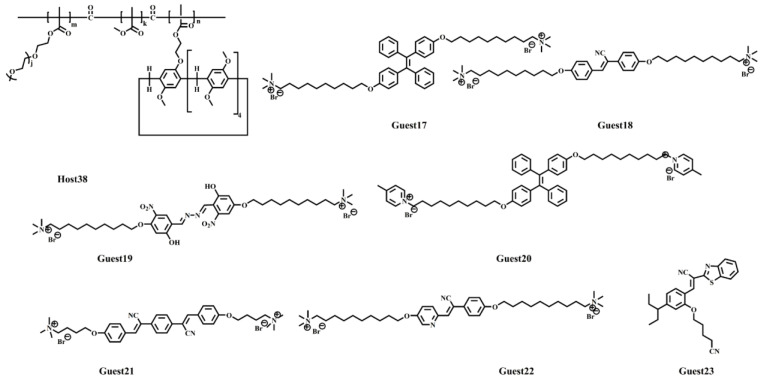
Molecular structures of pillar[*n*]arenes and guest molecules mentioned in this section.

The adjustability of supramolecular interactions, solvents, and competing substances with varying properties can influence and regulate the property of LHSs [185,186,187,188,189,190,191,192,193]. This section provides an overview of LHSs created by PSP-based materials. Some typical LHSs generated from PSP-based materials are summarized in Table 5. And the chemical structures of pillar[*n*]arenes and guest molecules mentioned in this section are listed in Scheme 6.

As shown earlier, different carrier platforms and energy transfer methods have been developed to emulate natural processes. To date, LHSs have made significant strides in achieving effective energy transfer from donor to acceptor. Nonetheless, most LHSs are synthesized in organic solvents, significantly restricting their utility in emulating natural LHSs. Supramolecular LHSs, assembled through non-covalent interactions, offer a solution by circumventing intricate synthesis and purification processes and showcasing remarkable AIE effects in aqueous surroundings. In the subsequent section, the development process of LHSs is extensively examined.

Currently, the antenna effects of the two-step sequential light collection systems remain modest. Consequently, designing and fabricating efficient sequential light collection systems in aquatic environments remains challenging.

**Figure 7:**
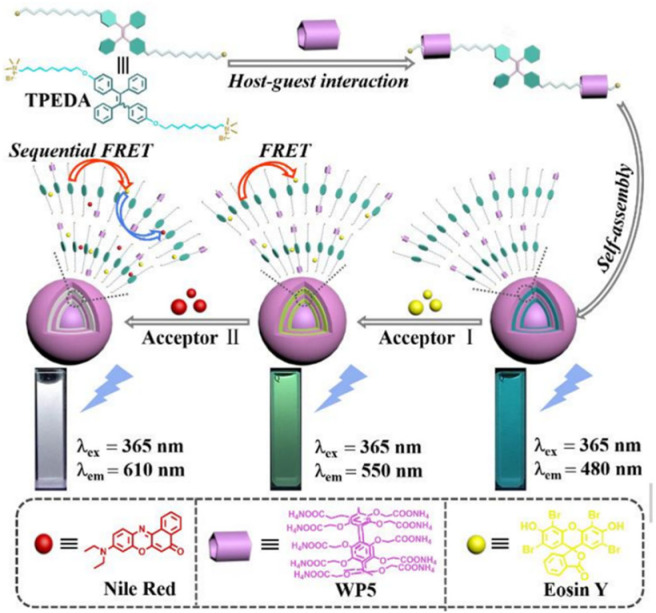
Schematic illustration of the self-assembly of pillar[5]arene-based aqueous light-harvesting system with two-step sequential energy transfer [194]. Copyright 2019, Wiley publishing.

As an illustrative case, in 2019, Wang et al. [194] successfully engineered efficient LHSs using a supramolecular approach (Figure 7). They achieved this structure by self-assembling supramolecular vesicles from supramolecular interaction complexes created with **Host8**, a bola-type tetraphenylethylene-functionalized dialkyl ammonium derivative, and two fluorescent dyes, Eosin Y (ESY) and Nile Red (NiR). They successfully achieved an efficient energy transfer from AIE **Guest17** to ESY. The WP5⊃TPEDA-ESY-NiR system proved to be a valuable nanoreactor for photocatalyzing the dehalogenation of *α*-bromoacetophenone in an aqueous medium, yielding an impressive 96 % yield.

One year later, the same research group [195] developed even more efficient LHSs using WP5⊃BPT in an aqueous environment. This was achieved through the supramolecular interactions between **Host8** and a bola-type bis(4-phenyl)acrylonitrile derivative **Guest18**. This resulted in an exceptionally strong antenna effect, with a donor/acceptor ratio reaching 350/1. By fine-tuning the molar ratio of DBT (4,7-bis(thien-2-yl)-2,1,3-benzothiadiazole) and NiR, it was possible to achieve vibrant white light emission, accompanied by an impressive fluorescence quantum yield of up to 23.5%. This material held significant potential for applications in visible light photocatalysis.

In 2021, Sun et al. further advanced the progress of LHSs [196]. They introduced two remarkably effective LHSs using a supramolecular approach involving a novel bola-type salicylaldehyde-azine derivative, and two distinct hydrophobic fluorescent dyes. These LHSs demonstrated a substantial antenna effect, characterized by donor/acceptor ratio of [HNPD]/[ESY] = 250/1 and [HNPD]/[NiR] = 200/1, respectively. The resulting WP5⊃HNPD (**Host8** and **Guest19**) proved to be exceptional energy donors. Solar energy was effectively harvested and converted into chemical energy, notably during the photocatalytic dehalogenation of *α*-bromoacetophenone. The energy output held promising potential for applications in mimicking natural photosynthesis systems.

Drawing from extensive prior research, Xiao et al. [197] pursued further investigations and established pillar[5]arene-mediated nanoparticles (**Host8** and **Guest20**) to attain a highly efficient LHS. These nanoparticles featured the integration of a hydrophobic dye, ESY, which served as a relay acceptor. With a swift and effective energy transfer process, the excitation energy exhibited an efficient transfer from the donor to the ultimate acceptor. Consequently, this material proved to be excellent for use in LED (light-emitting diode) devices.

Recognizing the significance of guest molecules in shaping the properties of assemblies, Xing et al. [198] devised and built efficient LHSs. This system was established on the supramolecular interactions between a cyano-substituted *p*-phenylenevinylene derivative **Guest21** and **Host8**. Two conventional fluorescent dyes were utilized as acceptors, facilitating a highly efficient two-step sequential energy transfer process, ultimately leading to energy transfer to the near-infrared region. The hydrophobic surroundings within the nanoparticles facilitated the aerobic cross-dehydrogenative coupling reaction in an aqueous environment. This research aimed to convert solar energy into chemical energy within the aqueous solution system.

In pursuit of exceptional binding capabilities and aqueous solubility, various types of pillar[5]arenes have been incorporated into LHSs. Also, a phosphate-**Host8** was engineered by Tang et al. [199]. The system comprised a phenyl-pyridyl-acrylonitrile derivative (PBT, **Guest22**), WPP5 (phosphate-**Host8**), and the organic pigment ESY. In water, WPP5 could bind with PBT, leading to the formation of WPP5⊃PBT complexes. These complexes were assembled into WPP5⊃PBT nanoparticles, exhibiting exceptional AIE properties. The antenna effect of the WPP5⊃PBT-ESY-based LHS was as high as 30.3. Energy transfer from PBT to ESY was observed, resulting in a notable increase in the absolute fluorescence quantum yields. WPP5⊃PBT-ESY-based LHSs were used as photosensitizers to catalyze the cross-coupling dehydrogenative (CCD) reaction between benzothiazole and diphenylphosphine oxide. 

**Figure 8:**
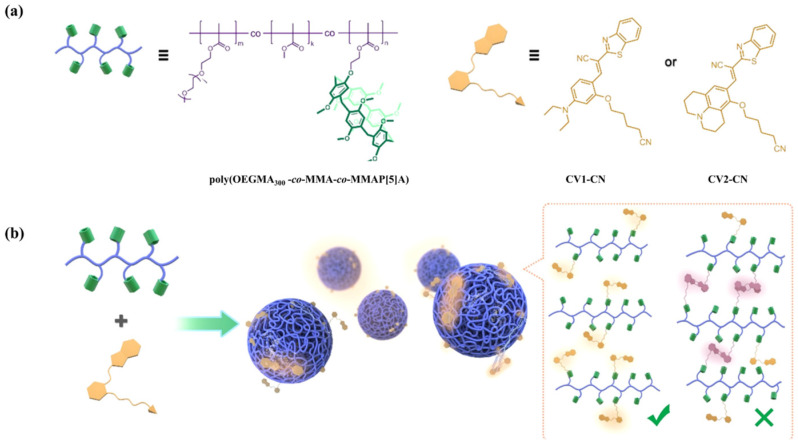
Schematic illustration of SPNs formed by CV derivatives and pillar[5]arene-based polymer. (**a**) Chemical structures of polymer and CV derivatives. (**b**) Formation of SPNs [200]. Copyright 2021, American Chemical Society.

In addition to previously indicated core-shell globular structures, innovative supramolecular polymer networks utilizing pillar[*n*]arene have proven valuable for building LHSs. As an illustration, Yang et al. [200] created a copolymer host material featuring pillar[5]arene units (**Host38**) as side chains (Figure 8). The cyanovinylene-based (CV) derivatives were integrated into the polymer structures. They hypothesized that the control of CV derivatives by amphiphilic polymer hosts would not only enhance the supramolecular aggregation-induced emission enhancement (SAIEE) system but also elucidate the operational mechanism of intramolecular motion, combining insights from both supramolecular interaction chemistry and polymer science. The copolymers could generate luminescent supramolecular polymer nanoparticles. These nanoparticles exhibited notable enhancements in emission and offered tunable fluorescence wavelengths. These improvements included the prevention of excimer formation within the CV moieties.

### 2.5. Artificial Nanochannel

**Scheme 7:**
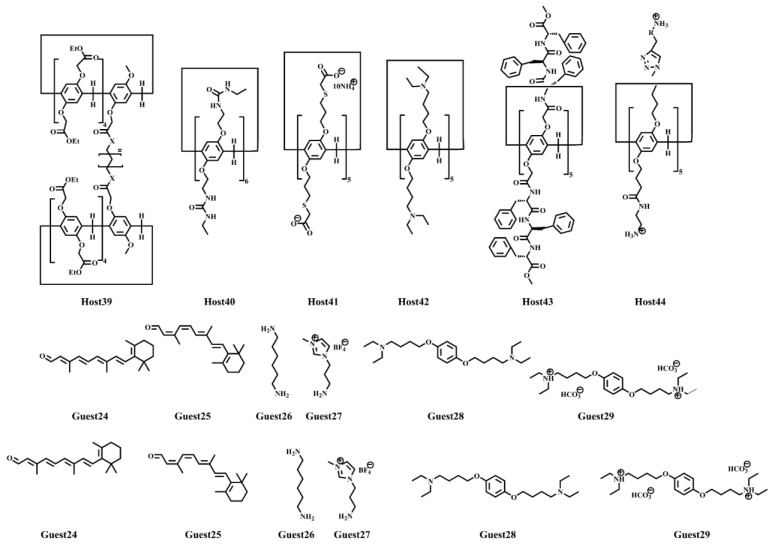
Molecular structures of pillar[*n*]arenes and guest molecules mentioned in this section.

Chemists have dedicated significant effort to constructing synthetic mimics inspired by the multifaceted functions of membrane proteins. This endeavor aims to gain insights into the transport mechanisms of membrane proteins, design separation materials, and develop therapeutic agents [201,202,203,204,205,206]. Cell membranes predominantly comprise phospholipids, and ions and polar molecules can only traverse them through specialized channels. Channel proteins are crucial in governing cellular processes, facilitating intercellular communication, and modulating the electrical excitability of nervous and muscle systems [207]. Consequently, chemists have maintained a sustained interest in creating artificial channels [208,209,210,211]. Within intricate self-assembly systems, the insertion of channels and subsequent transmembrane transport represent common dynamic processes [212,213,214,215]. As a next-generation channel, pillar[*n*]arene possesses robust transport capabilities owing to its symmetrical cavity structures [216]. Incorporating non-covalent interactions is anticipated to enhance the stability of its tubular conformation. So far, scientists have employed self-assembly methods to create a diverse array of synthetic transmembrane channels [217,218,219,220,221]. This section outlines the utilization of supramolecular columnar aromatics that aggregate in developing artificial nanochannels. The relevant research results are listed in Table 6. And the chemical structures of pillar[*n*]arenes and guest molecules mentioned in this section are listed in Scheme 7.

Nanochannels exhibit external stimuli responsiveness, encompassing light, temperature, and chirality. Since the pioneering work of Tabushi et al., who reported the use of alkylated cyclodextrin for Cu^2+^ and Co^2+^ transport in 1982, numerous researchers have been engaged in ongoing investigations, resulting in the synthesis of a wide range of artificial transmembrane channels.

In recent years, several channels have focused on K^+^. The work of Hou et al. [222] was instrumental in designing novel unimolecular artificial channels using pillar[6]arenes in 2015. Their approach involved extending the lengths of these channels by incorporating various ester, hydrazide, and short peptide chains. Pillar[6]arene was a rigid framework, while the appended chains could extend the tubular structures. The hydrazide and peptide chains were responsible for forming intramolecular N–H···O=C hydrogen bonds, leading to an enhancement in the overall tubular conformation of the molecule. The channel exhibited selectivity in transporting protons, water, and amino acids, demonstrating voltage-gated K^+^ transport. Aromatic hydrazide helices and macrocycles were crucial in mediating the selective transport of ammonium ions.

**Table 6:** Summary of artificial nanochannel materials described in this review.

Type of Pillar[*n*]arenes	Guest	Supramolecular Interactions	Diameter	Application	Ref.
**Host40**	**Guest24** **Guest25**	Host–guest	Larger opening: 450 nm Apex opening: 15 nm Contact angle: 55.8 ± 0.6°	Tunable and recycle transport of Cl^−^ (pH 3.78). Average transport rate: 5.578 ± 0.758 nmol/cm^2^·h (darkness); 0.721 ± 0.085 nmol/cm^2^·h (visible light).	[223]
**Host41**	**Guest26**	Host–guest	Large opening: 560 nm Tip: 20 nm Contact angle: 54.7 ± 0.6°	Tunable transport of K^+^ under the stimuli of Hg^2+^.	[36]
**Host8**	**Guest27**	Host–guest	Large opening: 450 nm Tip: 16 nm Contact angle: 34.6 ± 1.3°	Transport of K^+^ under the stimuli of temperature (pH 7).	[224]
**Host42**	**Guest28** **Guest29**	Host–guest	Nanochannel: 60 ± 10 nm Contact angle: 31.4 ± 2.5°	CO_2_/N_2_-active recycle transport of K^+^ (pH 5.5). Average transport rate: 1.66 × 10^−4^ nmol/cm^2^·h; 7.98 × 10^−4^ nmol/cm^2^·h.	[225]
**Host43**	−	−	Radius of liposome: 80 nm	Transport of H_2_O. Average transport rate: 1.3 × 10^9^ water molecules/s.	[226]
**Host44**	−	−		Transport of H_2_O. Stable flux: 42 L·m^−2^·h^−1^	[227]

Ion channels responsive to visible light play a vital role in metabolic processes. Research into these visible light-response membrane channels is significant for comprehending signal transduction in biological functions and their valuable applications in fostering interdisciplinary research endeavors. Li et al. [223] presented a clever chloride ion (Cl^−^) transport membrane channel that relied on supramolecular interactions. They harnessed a natural retinal chromophore (**Guest24**, **25**), which responds to visible light, as a guest molecule in their artificial channels. Ion transport was controllable through an ethyl-urea-derived pillar[6]arene (**Host40**), involving the threading or de-threading of the retinal and the selective binding of chloride ions. This system’s LOD was measured at 1.095 μM. The transporter has promising applications in natural photoelectric conversion and optical control transmission systems.

Li et al. [36] developed a new K^+^ channel. It is worth noting that mercury (Hg^2+^) is a highly toxic pollutant capable of causing harm to nerve tissue and organs. The binding of Hg^2+^ ions can block K^+^ channels, leading to in vivo toxicity. Hence, creating a straightforward and efficient artificial device to mimic the biological functions of Hg^2+^-gated K^+^ channels is crucial. Pillar[*n*]arene, a novel macrocyclic host, offers a simple and robust means to establish an “on-off” switch. Based on these considerations, this research group has been actively designing a tunable mercury(II) ion gate system regulated by mercaptoacetic acid-pillar[5]arene (**Host41**), employing biomimetic strategies. They successfully achieved reversible switching to potassium ion transport by controlling the presence of Hg^2+^ ions. The nanochannel demonstrated specific recognition of Hg^2+^, making it capable of distinguishing Hg^2+^ with a detection limit as low as 1 nanomolar. This research holds significant value for advancing the field of biological sciences.

Temperature-sensitive ion channels are vital components in numerous cellular processes. Incorporating supramolecular interactions involving pillar[*n*]arene can be applied to the nanochannels. Li et al. [224] pioneered the development of temperature-sensitive artificial ion nanochannels using pillar[5]arene (**Host8**), opening up new avenues in this field. The inspiration for this concept was drawn from biological temperature-sensitive channels. Through temperature stimulation within the range of 25 to 55 °C, the ion transport mechanism shifted from cations to anions, achieved by controlling the binding capacity based on supramolecular interactions. The observed outcome resulted from alterations in the nanochannel’s inner surface charge and wettability during the disassembly and assembly processes. These changes enabled precise and controlled biomimetic channel behavior.

Examples of gas-response nanochannels, particularly responsive to CO_2_, were relatively scarce. In 2021, Qin et al. [225] introduced solid-state nanochannels that enabled the controlled modulation of ion transport in response to alternating CO_2_/N_2_ stimuli. The pillar[5]arene with diethylamine groups (**Host42**) transformed to form cationic tertiary ammonium salt groups upon CO_2_ absorption. This modification rendered the ion nanochannels stable and repeatable for CO_2_ activation. 

Currently, various synthetic water channels have been created. Pillar[5]arene derivatives have proven to be excellent templates for generating tubular structures through functionalization. These structures have the potential to display specific water transport capability.

**Figure 9:**
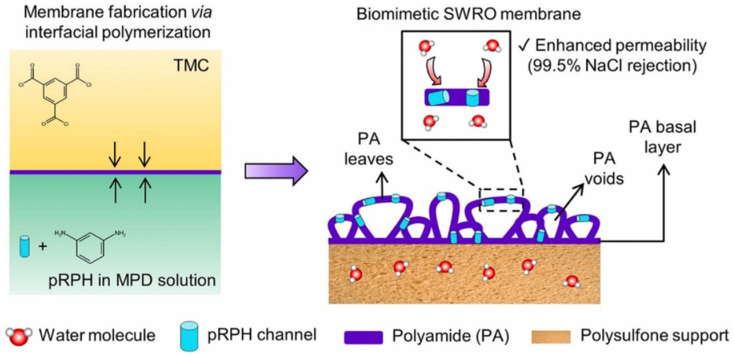
Incorporating pillar[5]arene water nanochannels into the polyamide selective layer [227]. Copyright 2022, Elsevier.

In 2019, Wang et al. [226] introduced peptide-attached pillar[5]arene (**Host43**) channels to mimic the high permeability of Aquaporins (AQPs) for purification applications. These channels facilitated smooth and efficient water transport, featuring an open pore. The channels possessed competitive water permeability compared to AQPs, showcasing their superior performance. When incorporated into a polymeric membrane, these channels demonstrated exceptional water-transport properties. This membrane significantly enhanced water flux while maintaining high salt rejection rates. In 2022, Lim et al. [227] investigated another type of biomimetic membrane based on nanochannels, which incorporated pillar[5]arene (**Host44**) water channels for seawater reverse osmosis desalination (Figure 9). The pillar[5]arene channels were incorporated into the layer of seawater reverse osmosis (SWRO) membranes using interfacial polymerization. The optimized membrane achieved a water permeability of 2.52 L∙m^−2^∙h^−1^∙bar^−1^ and a rejection rate of 99.5%.

### 2.6. Drug Delivery System

**Scheme 8:**
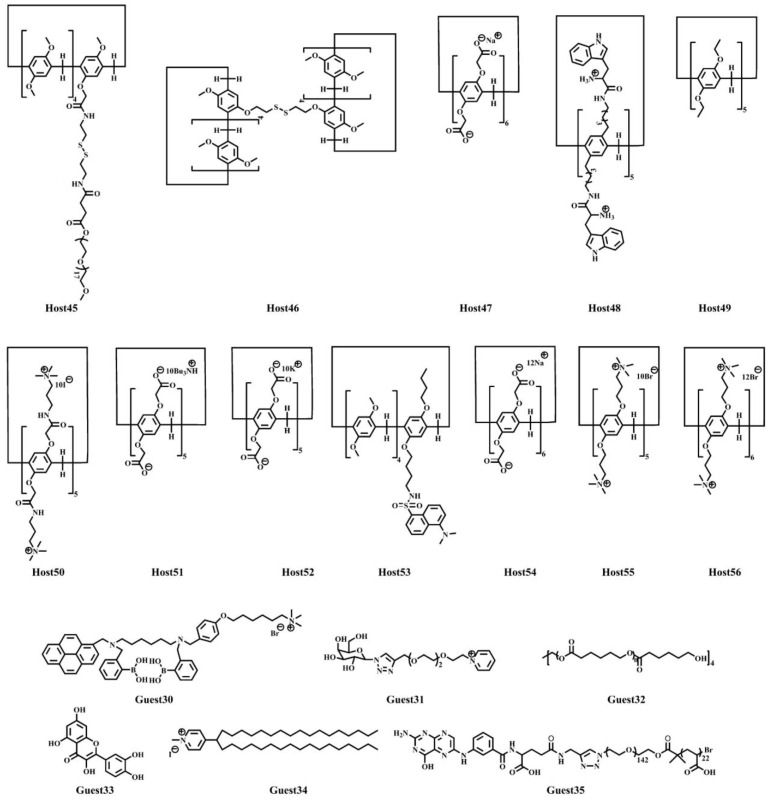
Molecular structures of pillar[*n*]arenes and guest molecules mentioned in this section.

Polysaccharide-polyphenol supramolecules, characterized by their distinctive skeleton structures and supramolecular properties, exhibit remarkable qualities such as biocompatibility, degradability, compatibility with other substances, and self-adaptability [228,229]. Pillar[*n*]arene, known for its straightforward synthesis and purification processes, has demonstrated impressive performance in various biomedical applications [230,231,232,233]. However, it is important to note that inserting cancer drugs into DNA can lead to cytotoxicity [234,235]. Utilizing pillar[*n*]arene-based components allows for the incorporation of different functional groups, enabling the controlled release of the encapsulated substances. This strategy can achieve the desired outcome, reducing biological toxicity [236,237,238]. Supramolecular interactions are promising for achieving site-specific drug or biomolecule release and addressing biological barriers [239,240,241,242,243,244,245]. The following section presents several drug delivery systems based on pillar[*n*]arene derivatives. These drug delivery systems are categorized based on the structures of pillar[*n*]arene and the specific stimuli conditions, as depicted in Table 7. And the chemical structures of pillar[*n*]arenes and guest molecules mentioned in this section are listed in Scheme 8.

In 2019, Yang et al. and Wang et al. independently developed distinct glutathione (GSH)-responsive drug delivery systems based on pillar[5]arene (**Host45**). Yang et al. [246] designed a novel amphiphilic pillar[5]arene-based pseudo[1]rotaxane (PPR) containing a disulfide bond in the self-included linker. This PPR was then used as the hydrophobic core, covalently linked to biocompatible PEG (polyethylene glycol). The PPR molecules could self-assemble, forming vesicles with excellent colloidal stability in an aqueous environment. When exposed to a high glutathione concentration, the vesicles decomposed due to the breakage and exchange of disulfide bonds. This vesicular system efficiently encapsulated the anti-cancer drug doxorubicin (DOX) with a high loading capacity and facilitated controlled drug delivery without inducing cell cytotoxicity. The same year, Wang et al. [247] introduced nanoparticles loaded with paclitaxel (PTX) that exhibited dual responsiveness to stimuli. These nanoparticles were based on a bispillar[5]arene (**Host46**) and were triggered by spermine and GSH near cancer cells. This design allowed for precise and selective drug release within lung cancer cells, resulting in specific antitumor activity against these cancer cells. Macrocyclic molecules have been employed to create innovative nanostructures, contributing to the advancement of biomaterials and pharmaceuticals.

Numerous glucose-responsive systems have emerged as drug delivery systems. For instance, Zuo et al. [248] introduced a multi-responsive insulin delivery supramolecular platform that self-assembles using pillar[5]arene **Host47** and a diphenylboronic acid derivative **Guest30**. These vesicles served as both glucose sensors and closed-loop insulin delivery actuators. They could simultaneously encapsulate insulin and glucose oxidase. In a hyperglycemic environment characterized by low pH and high levels of H_2_O_2_, the vesicles underwent rapid degradation, facilitating the efficient release of insulin. The nanocarrier, which exhibited excellent cytocompatibility, responded promptly to hyperglycemic conditions and effectively regulated glucose levels within the normal range. 

Vesicles demonstrated remarkable pH responsiveness and rapid drug release in acidic environments. Pei et al. [249] pioneered a targeted drug delivery system that binds with DNA. This system was based on supramolecular vesicles, which self-assembled through the supramolecular interactions between TP5 (**Host48**) and **Guest31**. The DOX-loaded TP5G vesicles displayed exceptional pH responsiveness, resulting in the rapid release of DOX within an acidic environment. Moreover, DOX-loaded TP5G exhibited a hepatoma-targeting capability towards HepG2 cells and demonstrated significantly enhanced cytotoxicity against hepatoma cells, including DOX-resistant variants. This increased cytotoxicity was attributed to the substantial interactions between the Trp-rich segments in TP5 and DNA within the cells.

Shi et al. [250] achieved the successful synthesis of a nonionic polyrotaxane (PR) based on pillar[5]arene DEP5 (**Host49**). This PR was composed of poly(*β*-caprolactone) and adamantane. Notably, the crystallinity of the PR experienced a significant decrease. They also went on to construct a pseudoblock polymer. PR-PAA combines PR with CD-PAA (*β*-cyclodextrin end-capped pH-stimulated poly(acrylic acid)). The supramolecular polymer could self-assemble, resulting in the formation of vesicular nanoparticles based on PR-PAA. The drug loading capacity was improved due to cavities at DEP5 and the multi-branched structures of PR and CD-PAA. These vesicles could accumulate in tumor tissues owing to the enhanced permeability and retention (EPR) effect. Under acidic conditions, drugs could be selectively released, potentially enhancing cellular uptake and reducing cytotoxicity against SMMC-7721 cells.

In 2022, Stoikov et al. [251] and Yan et al. contributed to investigating novel drug delivery systems for encapsulating different drugs. Stoikov’s team introduced a novel interpolyelectrolyte non-toxic complex, which is self-assembled using water-soluble pillar[5]arenes (**Host50, 51, 52**). The spherical aggregates demonstrated an average diameter of approximately 10 nm. The interpolyelectrolyte complex is efficiently bound to quercetin. The release rate of quercetin from the complex was investigated in various acid solutions. Quercetin remained unreleased and unoxidized at pH 4, which has implications for developing multi-target drugs. Yan et al. [252] also designed a sensitive fluorescent probe called 4C-G based on the intramolecular charge transfer (ICT) mechanism. This probe exhibited favorable sensitivity to various types of proteins. Dansylamide was found to have an affinity for binding with pillar[5]arene (**Host53**), and they self-assembled into a complex. The presence of **Host53** facilitated the insertion of dansylamide into proteins, particularly bovine serum albumin. The 4C-G-REG complexes assembled into fluorescent nanoparticles with a high drug loading capability at pH 7.4. The fluorescence intensity increased when the pH was shifted to acidic conditions (pH 6.0). This probe could be applied for imaging HepG2 cells and serve as a drug carrier with anti-cancer activity.

As our understanding of pillar[5]arene has deepened, there has been a gradual shift in attention towards other pillar[*n*]arenes, particularly pillar[6]arene. In 2014, Wang et al. [253] developed supramolecular binary vesicles using supramolecular complexation involving **Host54**, which is soluble in water, and the SAINT molecule (**Guest34**). These vesicles were designed for bioimaging purposes. These supramolecular vesicles exhibited responsiveness to changes in pH, Ca^2+^, and temperature. They efficiently encapsulated calcein, and when the solution’s pH was adjusted to acid levels or when calcium ions were introduced, calcein could be efficiently released from the vesicles. The supramolecular vesicles effectively encapsulated DOX and exhibited efficient drug release in response to variations in pH or the presence of calcium ions. This controlled released mechanism reduced the toxicity of the drug to normal cells. By raising the temperature of the vesicle solution containing WP6 and SAINT, supramolecular vesicles with a large internal volume and excellent stability could be obtained. 

**Figure 10:**
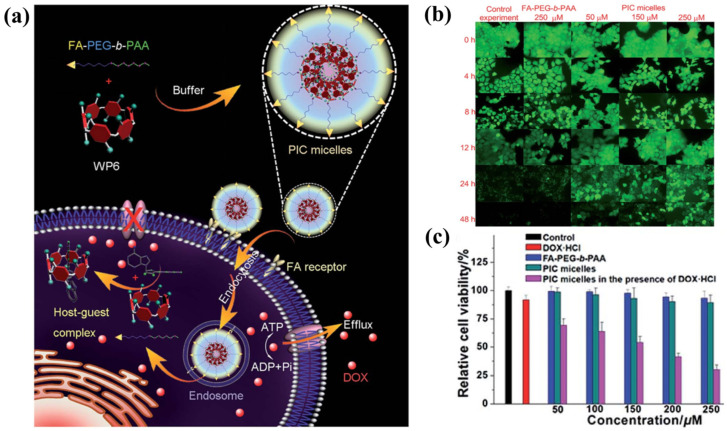
(**a**) Schematic illustration of the preparation of PIC micelles and possible mechanism to inhibit the efflux pump in the cell. (**b**) Fluorescence images of the MCF-7/ADR cells stained with calcein-AM. Cells were incubated without/with FA-PEG-*b*-PAA, PIC micelles containing different amounts of WP6. (**c**) Cytotoxicity of DOX·HCl, FA-PEG-*b*-PAA, PIC micelles, and DOX·HCl-loaded PIC micelles with different concentrations of WP6 against MCF-7/ADR cells [254]. Copyright 2016, The Royal Society of Chemistry.

In 2016, Huang et al. [254] employed the supramolecular complexation (polyion complex (PIC) micelles) between WP6 (**Host55, 56**) and **Guest35** to inhibit ATP (adenosine triphosphate) hydrolysis effectively (Figure 10). They also used a folic acid-functionalized diblock copolymer (FA-PEG-*b*-PAA) to impart specific targeting capability to the PIC micelles. These micelles were designed to deliver WP6 to KB cells that over-express folate receptors preferentially. This supramolecular complexation approach was employed to inhibit the efflux pumps that used ATP to transport anti-cancer drugs (i.e., DOX) out of cells, effectively cutting off their energy source. This strategy enhanced the efficacy of cancer chemotherapy.

## 3. Conclusions and Outlook

This review summarizes the advancements in utilizing PSP-based materials. Over the past decade, significant strides have been achieved in this field, showing promising progress. Numerous endeavors have been dedicated to supramolecular polymers formed through supramolecular interactions involving pillar[*n*]arene. These efforts have allowed for the manipulation of self-assembly properties and have set numerous precedents for future research to optimize various attributes of PSPs.

Due to the persistent efforts of researchers in the last decade, this novel category of PSP materials has found applications across various domains. In sensor development, especially fluorescence sensors, functionalized pillar[*n*]arenes and guest molecules have been employed to create supramolecular polymers with diverse structures through non-covalent interactions. This has enabled the highly sensitive identification and detection of specific harmful substances. In terms of environmental science and industry, these polymer materials have proven to be effective in adsorbing various substances such as organic dyes, metal ions, and toxic gases, serving purification purposes. Importantly, many of these materials are designed with recyclability in mind, ensuring that performance remains uncompromised. Pillar[5]arene can engage in interactions with various metal nanoparticles, leading to the formation of aggregates with diverse structures. These interactions have been instrumental in achieving high catalytic efficiencies in environmentally friendly industrial processes. Researchers have developed LHSs to tackle the energy crisis by incorporating pillar[5]arene with fluorescent dye molecules like ESY and NiR. This approach enhances Forster Resonance Energy Transfer (FRET) efficiency, antenna effects, and fluorescence quantum yield, resulting in highly satisfactory outcomes. In the biomedical sector, the channel-switching behavior of pillar[*n*]arenes can be precisely regulated by adjusting their binding capacities based on supramolecular interactions, which is influenced by external stimuli like temperature, light, gas, and ions. Stimuli-responsive nanoparticles utilizing pillar[*n*]arene, responsive to factors such as GSH, pH, temperature, and ions, offer means for precise drug delivery and release. These materials boast advantages, including low toxicity, high efficiency, and stability. Given these accomplishments, researchers must foster collaborative efforts to study and develop high-performance supramolecular polymer materials.

While functional pillar[*n*]arene holds promising application prospects across various domains, it is important to acknowledge that certain issues and challenges still need to be addressed and overcome in their utilization. One of the primary challenges lies in the design and synthesis of macrocyclic molecular structures like functionalized pillar[*n*]arenes. Achieving high yields and purified products can be challenging for this synthesis process. Developing more efficient techniques for synthesizing functionalized pillar[*n*]arene is essential. Additionally, while pillar[5]arene has been extensively studied, studies on higher pillar[*n*]arenes with larger cavities (*n* ≥ 6) remain scarce. Varying cavity sizes can introduce novel characteristics to pillar[*n*]arenes. Consequently, there is significant research potential in designing and synthesizing different functional pillar[*n*]arene materials and investigating their practical applications. Furthermore, the morphological distinctions observed in PSPs (such as irregular spherical, three-dimensional network, vesicular structure, etc.) are intricately linked to the properties of these materials. Diverse cavity sizes can introduce fresh attributes to pillar[*n*]arenes. Synthesizing polymer materials based on pillar[*n*]arenes with topological structures using a straightforward and efficient approach is a valuable endeavor. A wide range of potential applications can be realized by modifying the pillar[*n*]arene framework with diverse functional units and encapsulating different small molecules or innovative two-dimensional techniques for tailored self-assembly. These applications encompass molecular machinery, supramolecular liquid crystals, magnetic resonance imaging, and antibacterial materials. Regarding fluorescence detection, there is a scarcity of reports on emerging PSP sensors capable of simultaneously detecting multiple analytes based on current research. While chemists have developed numerous macrocyclic hosts in substance adsorption and separation, only a few cross-linked polymers have found effective applications in pollutant treatment. In the biomedical domain, pillar[*n*]arene, a significant macrocyclic molecule characterized by its cavity structure, shares similarities with other macrocyclic molecules regarding protein recognition. However, there is currently a scarcity of investigations concerning the interactions between pillar[*n*]arene and proteins. Pillar[*n*]arene has potential applications in the treatment of certain epidemic viruses. Modifying specific targeting units makes identifying and capturing essential proteins implicated in virus replication feasible, ultimately leading to antiviral effects. 

Considering the invaluable application prospects of novel PSP-based materials for societal advancement and human well-being, the development of PSPs in polymer chemistry is expected to progress much faster than anticipated. We aspire for this review to stimulate the development of intelligent PSP materials with novel structures and functionalities in the future, contributing to scientific and technological advancements.

## Figures and Tables

**Table 1:** Previous reviews on designs and applications of pillar[*n*]arene-based supramolecular polymers.

Focused Pillar[*n*]arenes	Applications	Ref.
Pillar[5]arene Pillar[6]arene	Sensor	[20]
Pillar[5]arene	Sensor	[38]
Pillar[6]arene	Sensor; substance adsorption and separation	[57]
Pillar[5]arene pillar[6]arene	Sensor; substance adsorption and separation; drug delivery system	[58]
Pillar[5]arene Pillar[6]arene	Sensor; catalysis; light-harvesting system; drug delivery system	[59]
Pillar[5]arene Pillar[6]arene	Sensor; catalysis; light-harvesting system; biomedical	[60]
Pillar[5]arene	Artificial nanochannel	[37]
Pillar[5]arene	Artificial nanochannel	[61]
Pillar[5]arene Pillar[6]arene	Biomedical	[62]
Pillar[5]arene Pillar[6]arene	Substance adsorption and separation; light-harvesting system; artificial nanochannel; drug delivery system	[63]
Pillar[5]arene Pillar[6]arene	Biomedical	[64]
Pillar[5]arene Pillar[6]arene	Fluorescence sensor; substance adsorption and separation; catalysis; light-harvesting system; artificial nanochannel; drug delivery system	This work

**Table 3:** Summary of adsorption and separation materials described in this review.

Type of Pillar[*n*]arenes	Guest	Supramolecular Interactions	Morphology	Application	Ref.
**Host12**	−	Host–guest	Spherical/rodlike	Removal of environmentally polluted and security concerned chemicals and targets.	[120]
**Host13**	**Guest6, 7, 8, 9**	Host–guest	Crystal	Selective and stable removal of heterocyclic impurities from toluene (purity: 99%).	[121]
**Host14**	−	Host–guest	1D channel (6.7 Å)	Removal of gases and vapors of saturated hydrocarbons.	[122]
**Host15**	−	Hydrogen bond	3D network	Superior removal of organic micropollutants from water (removal efficiency: 17.0−99.8%).	[123]
**Host16** **Host17**	−	Host–guest	3D network	Recycle adsorption, removal in organic micropollutants (removal efficiency: 30.1−91.5%).	[124]
**Host18**	−	Host–guest	Irregular nanometer- to micrometer-sized nonporous particulates	Recycle removal of low-molecular-weight organic acids, amines, alcohols from water (removal efficiency: 47.5−98.5%).	[125]
**Host19**	**Guest5**	Host–guest	Line	Rapid removal of paraquat (removal efficiency: 90%).	[126]
**Host20**	**Guest5**	Host–guest	Porous	Efficient removal of paraquat (removal efficiency: 98.40%).	[127]
**Host21**	**Guest5**	Host–guest	Uniformly distributed crystal	Removal of paraquat from water (adsorption efficiency: 98%).	[128]
**Host17** **Host22**	−	Host–guest, π–π	2D	Ultrasensitive adsorption of multianalytes, including Fe^3+^, Hg^2+^, Ag^+^, F^−^, and Br^−^ (adsorption rates: 90.12−99.95%).	[129]
**Host23**	−	Host–guest, π–π	Spherical	Recycle removal of mercury(II) (removal efficiency: 90.00%).	[130]
**Host23**	**Guest10**	Host–guest, π–π	1D channel	Recycle separation of bromoalkanes isomers.	[131]
**Host24**	**Guest11**	Host–guest, π–π	Daisy chain	Recycle CO_2_ capture and iodine adsorption (removal efficiency: 94%).	[132]
**Host25**	−	−	Nanometer-sized porous structure with voids	Adsorption of iodine from water and vapor phases (removal efficiency: 95%).	[133]
**Host26**	−	Host–guest	−	Separation of propane/methane.	[134]

**Table 4:** Summary of catalysis materials described in this review.

Type of Pillar[*n*]arenes	Other Components	Supramolecular Interactions	Morphology	Catalyzed Reaction	Ref.
**Host27**	**Guest12**	π–π, Hydrogen bond	Stacked lamellar	Dehalogenation reaction.	[154]
**Host28**	**Guest13**	Host–guest, π–π	Multilayer nanosheets	Photocatalytic cross-coupling hydrogen evolution reaction.	[155]
**Host29**	**PdNPs**	Host–guest, Coordination	Cross-linked polymer with irregular spherical shape (105 nm)	Suzuki–Miyaura coupling reaction.	[156]
**Host30**	**Guest14**	Host–guest, Coordination	Disperse well with high density (9 nm)	Suzuki–Miyaura coupling reaction. Nitrophenol reduction.	[157]
**Host31**	**PdNPs**	Coordination, C–H···π	Hinges by small particles (50 nm)	Suzuki–Miyaura coupling reaction.	[158]
**Host32**	**Guest15**	Host–guest, Coordination	3D networks	Reductions of toxic nitroaromatics Suzuki–Miyaura coupling reaction.	[159]
**Host33**	**Guest1**	Host–guest, Hydrogen bond	Fiber (100 mmol) Three-dimensional network (200 mmol)	Fenton-like reaction in water.	[160]
**Host34**	**AuNPs**	Host–guest	Vesicles, nanotubes, 1D/2D	4-nitrophenol reaction in water.	[161]
**Host34**	**Guest16**	Host–guest	AuNPs (10 nm) uniformly dispersed on the surface of SWCNT	Ethanol oxidation reaction in water.	[162]
**Host35**	**Guest5**	Host–guest	Spherical AgNPs dispersed on the surface of SWCNT and located at sites of **Host35**	Degrading organic dye molecule in water.	[163]
**Host36**	**Guest5**	Host–guest	COF: 2D structure with spherical AgNPs (6–7 nm)	Reaction of paraquat in water.	[164]
**Host37**	**Polyoxometalates**	Host–guest	Spherical	Recycle catalytic oxidation of aldehydes into carboxylic acids.	[165]

**Table 5:** Summary of light-harvesting systems described in this review.

Type of Pillar[*n*]arenes	Guest	FL Probe	Supramolecular Interactions	Morphology	Property	Ref.
**Host8**	**Guest17**	ESY, NiR	Host–guest	Spherical	Electrode potential: 19.84 mV. *λ*_ex_/*λ*_em_: 365 nm/610 nm. FRET efficiency: 56.28%. Antenna effect: 3.5. FL quantum yield: 5.01%.	[194]
**Host8**	**Guest18**	DBT, NiR	Host–guest	Multilayered spherical, core-shell	Electrode potential: 46.94 mV. *λ*_ex_/*λ*_em_: 365 nm/620 nm. FRET efficiency: 60.9, 89.4%. Antenna effect: 20.01. FL quantum yield: 23.5%.	[195]
**Host8**	**Guest19**	ESY, NiR	Host–guest	Core-shell, globular	Electrode potential: −23.41 mV. *λ*_ex_/*λ*_em_: ESY: 365 nm/550 nm; NiR: 365 nm/630 nm. FRET efficiency: 80.5, 60.2%. Antenna effect: 32.5, 30.1. FL quantum yield: 12.43, 15.24%.	[196]
**Host8**	**Guest20**	ESY, Sulforhodamine 101	Host–guest	Spherical	Electrode potential: 25.2 mV. *λ*_ex_/*λ*_em_: 330 nm/606 nm. FRET efficiency: 94%. Antenna effect: 24. FL quantum yield: 36.37%.	[197]
**Host8**	**Guest21**	ESY, NiR	Host–guest	Spherical	Electrode potential: −20.3 mV. *λ*_ex_/*λ*_em_: 365 nm/650 nm. FRET efficiency: 67%, 66%. Antenna effect: 11.5, 7.2. FL quantum yield: 8.7%.	[198]
**Host8**	**Guest22**	ESY	Host–guest	Spherical	Electrode potential: 28.7 mV. *λ*_ex_/*λ*_em_: 365 nm/552 nm. FRET efficiency: 67.5%. Antenna effect: 30.3. FL quantum yield: 35.7%.	[199]
**Host38**	**Guest23**	NiR	Host–guest	Spherical	*λ*_ex_/*λ*_em_: 460 nm/520 nm. FRET efficiency: 76.41%. FL quantum yield: 5.68%.	[200]

**Table 7:** Summary of drug delivery systems described in this review.

Type of Pillar[*n*]arenes	Other Components	Morphology	Encapsulation	Stimuli	Ref.
**Host45**	−	Hollow vesicles	Doxorubicin (DOX)	Glutathion (GSH)	[246]
**Host46**	−	Spherical (180 nm)	Paclitaxel (PTX)	GSH, spermine	[247]
**Host47**	**Guest30**	Vesicles	Insulin	Glucose	[248]
**Host48**	**Guest31**	Vesicles	DOX	pH	[249]
**Host49**	**Guest32**	Vesicles	DOX	pH	[250]
**Host50, 51, 52**	**Guest33**	Spherical (10 nm)	Quercetin	pH	[251]
**Host53**	−	Spherical (149.6 nm)	Regorafenib (REG)	pH	[252]
**Host54**	**Guest34**	Vesicles	DOX	Thermal, pH, Ca^2+^	[253]
**Host55, 56**	**Guest35**	Polyion complex micelles	DOX	−	[254]

## Abbreviations

Aquaporins	AQPs
Adenosine triphosphate	ATP
Aggregation-induced emission	AIE
4,7-bis(thien-2-yl)-2,1,3-benzothiadiazole	DBT
Cyanovinylene-based	CV
Cross-coupling dehydrogenative	CCD
Covalent organic framework	COF
Doxorubicin	DOX
Eosin Y	ESY
Enhanced permeability and retention effect	EPR
Forster Resonance Energy Transfer	FRET
Glutathione	GSH
Intramolecular charge transfer	ICT
Light-emitting diode	LED
Light-harvesting systems	LHSs
Limit of detection	LOD
Nile Red	NiR
Paclitaxel	PTX
Paraquat	PQ
Photoinduced electron transfer	PET
Pillar[*n*]arene-based supramolecular polymers	PSPs
Polyrotaxane	PR
Polyion complex	PIC
Polyethylene glycol	PEG
Quantum dots	QDs
Regorafenib	REG
Supramolecular aggregation-induced emission enhancement	SAIEE
Supramolecular organic frameworks	SOFs
Supramolecular polymers	SPs
Supramolecular polymer nanoparticles	SPNs
Single-walled carbon nanotubes	SWCNTs
Seawater reverse osmosis	SWRO
Two-photon fluorescent	TPF
Up conversion nanoparticles	UCNPs
